# Enhancing Stability and Mucoadhesive Properties of Chitosan Nanoparticles by Surface Modification with Sodium Alginate and Polyethylene Glycol for Potential Oral Mucosa Vaccine Delivery

**DOI:** 10.3390/md20030156

**Published:** 2022-02-22

**Authors:** Muhammad Khairul Amin, Joshua Siaw Boateng

**Affiliations:** School of Science, Faculty of Engineering and Science, University of Greenwich at Medway, Central Avenue, Chatham Maritime, Chatham, Kent ME4 4TB, UK; khairulsub@gmail.com

**Keywords:** chitosan, mucosal vaccination, mucoadhesion, nanoparticles, ovalbumin, polyethylene glycol, sodium alginate

## Abstract

**Background:** The present study aimed to fabricate surface-modified chitosan nanoparticles with two mucoadhesive polymers (sodium alginate and polyethylene glycol) to optimize their protein encapsulation efficiency, improve their mucoadhesion properties, and increase their stability in biological fluids. **Method:** Ionotropic gelation was employed to formulate chitosan nanoparticles and surface modification was performed at five different concentrations (0.05, 0.1, 0.2, 0.3, 0.4% *w*/*v*) of sodium alginate (ALG) and polyethylene glycol (PEG), with ovalbumin (OVA) used as a model protein antigen. The functional characteristics were examined by dynamic light scattering (DLS), X-ray diffraction (XRD), Fourier-transform infrared spectroscopy (FTIR), differential scanning calorimetry (DSC), and scanning electron microscopy (SEM)/scanning transmission electron microscopy (STEM). Stability was examined in the presence of simulated gastric and intestinal fluids, while mucoadhesive properties were evaluated by in vitro mucin binding and ex vivo adhesion on pig oral mucosa tissue. The impact of the formulation and dissolution process on the OVA structure was investigated by sodium dodecyl-polyacrylamide gel electrophoresis (SDS-PAGE) and circular dichroism (CD). **Results:** The nanoparticles showed a uniform spherical morphology with a maximum protein encapsulation efficiency of 81%, size after OVA loading of between 200 and 400 nm and zeta potential from 10 to 29 mV. An in vitro drug release study suggested successful nanoparticle surface modification by ALG and PEG, showing gastric fluid stability (4 h) and a 96 h sustained OVA release in intestinal fluid, with the nanoparticles maintaining their conformational stability (SDS-PAGE and CD analyses) after release in the intestinal fluid. An in vitro mucin binding study indicated a significant increase in mucin binding from 41 to 63% in ALG-modified nanoparticles and a 27–49% increase in PEG-modified nanoparticles. The ex vivo mucoadhesion showed that the powdered particles adhered to the pig oral mucosa. **Conclusion**: The ALG and PEG surface modification of chitosan nanoparticles improved the particle stability in both simulated gastric and intestinal fluids and improved the mucoadhesive properties, therefore constituting a potential nanocarrier platform for mucosal protein vaccine delivery.

## 1. Introduction

The human mucosa is the first line of defense against pathogenic infection and is continuously exposed to external assault from various microorganisms (bacteria, viruses). Infection may occur when the mucosal barrier is compromised [1]. Most vaccines are delivered by parenteral injections, which are generally poor in inducing mucosal immune responses and are therefore less effective against infection on mucosal surfaces [2]. Moreover, mucosal vaccines offer numerous advantages compared with parenteral vaccines, not only from a production or regulatory perspective, but also with regard to ease of administration, improved compliance, better and more practical mass vaccination campaigns, and a reduced risk of spreading blood-borne infections from contaminated injection needles [3]. In general, effective mucosal vaccine development targets the oral, nasal, and vaginal routes.

Indeed, the mucosal sites are anatomically adjacent and can be exploited to achieve effective immune responses. For example, immune responses are induced in both rectal and genital sites in response to rectal mucosa vaccine delivery, immune responses in Peyer patches (PPs) in the small intestine can be induced following oral immunization, and tonsil and adenoid immune responses can be induced by nasal or sublingual immunization [3,4]. However, the development of oral antigen (e.g., proteins and immune-induced agents) -loaded delivery systems targeting mucosal sites is significantly challenging for pharmaceutical scientists due to the acid and enzymatic degradation that takes place in the stomach and intestines, first-pass metabolism, the poor permeability across the gastrointestinal mucosa membrane, and the poor cellular uptake at the mucosal site [5]. To overcome these limitations, various strategies have been employed to deliver antigen cargo through mucosal sites in recent decades, including liposomes, nanoparticles, and nanoemulsions. Among these strategies, the use of natural biodegradable polymeric nanocarriers has gained significant interest. Such polymeric nanocarriers can act as platforms for targeted protein antigen delivery and have attracted the attention due to their versatility, ability to target tissues, and controlled release properties [6].

Both synthetic and natural polymers have been widely investigated with regard to developing a suitable protein delivery carrier in terms of their biodegradability, biocompatibility, low toxicity, and high level of protein entrapment [7]. Amongst all the polymers used for this purpose, chitosan has gained significant attention due to its excellent stability in biological media, biodegradable nature, low toxicity, mucoadhesive properties, enhanced penetration capacity across biological barriers, and good compatibility with protein molecules [8]. At physiological pH (6 to 7.5), chitosan possesses OH and NH_2_ groups that give rise to hydrogen bonding opportunities. The single-layered epithelial membrane found in the small and large intestines contains goblet cells which secrete mucus directly on the epithelial surfaces. The major component of mucus is mucin, and the hydrogen bonding of chitosan with mucin is likely to be the main reason why chitosan is considered a potential mucoadhesive vaccine delivery vehicle [9]. Since 1990, chitosan has been studied as a drug delivery material for controlled drug release and as a polymer matrix with the ability to temporarily open intracellular epithelial tight junctions and facilitate the uptake of hydrophilic drugs [10].

However, in recent years several research groups have investigated the formulation of chitosan nanoparticles as a protein delivery carrier to protect entrapped proteins from enzymatic degradation in biological systems, especially for oral delivery [11,12]. One of the major advantages of chitosan is its ability to form positively charged particles under mild agitation conditions without the need for harmful organic solvents. It can also facilitate the adsorption or encapsulation of therapeutic proteins and antigens or form polyplexes by electrostatic interaction with negatively charged nucleotides [13].

However, despite these advantages of chitosan, major challenges remain, owing to the wide range of physiological pH in the stomach and intestinal environment, with chitosan losing its capacity to enhance drug permeability and absorption. Further, the pKa of the primary amine group of chitosan is approximately 6.5 and it carries no charge at neutral pH; hence, it is insoluble in water [14]. The poor solubility of chitosan might impede antigen delivery at neutral pH environments. Therefore, the chemical modification of chitosan nanoparticles’ surfaces by introducing hydrophilic groups such as hydroxyalkyls, carboxyalkyls, succinyls, and thiols or by grafting polymers such as polyethylene glycol (PEG) or sodium alginate (ALG) would make it possible to retain the OH and NH_2_ groups of chitosan and protect the nanoparticles from the hostile environment of the gastrointestinal tract (GIT). [15].

Moreover, most vaccines on the market are liquid formulations, leading to some challenges such as cold-chain transportation and storage requirements, with resulting short shelf lives [16,17]. For example, influenza subunit vaccine showed an increased degradation rate of hemagglutinin when the storage temperature was increased from 5 °C to 25 °C [18], reflecting a change from cold temperate to hot tropical climates. Therefore, it is necessary to develop solid-state vaccine formulations using platforms such as microneedle patches or lyophilized matrices that have better stability during transportation and long-term storage as well as being more economical to distribute. The advantage of lyophilized matrices is that they can be reconstituted to different oral solid dosage forms such as tablets.

In this study, surface-modified chitosan nanoparticles have been designed to target the intestinal immune inductive site—i.e., the PPs—through vaccine delivery via the oral route. This is expected to protect nanoparticles from the harsh GIT environment and target the M-cells located in the PPs. Optimized chitosan nanoparticles loaded with ovalbumin (OVA) as a model protein-based immunogenic antigen were modified with ALG and (PEG). This will ensure their better stability, allowing higher amounts of the loaded antigen to reach the site of uptake (M cells in the PPs), improve their mucoadhesive properties, and improve the sustained release capability of the protein-loaded nanoparticles. OVA has been widely utilized as a model antigen in vaccine delivery research due to its convenient stability at room temperature and cost effectiveness [19]. ALG is a well-known gel-forming agent with strong mucoadhesive properties. Though PEG is not considered a typical mucoadhesive polymer, it has been shown to enhance mucoadhesion properties when copolymerized with other mucoadhesive polymers [20]. PEG can form a strong phase transitional shielding layer around chitosan nanoparticles to enhance stability in the GIT [15].

## 2. Results and Discussion

### 2.1. Particle Analyses Using Dynamic Light Scattering (DLS)

#### 2.1.1. Effect of Ovalbumin (OVA) Loading

In this study, ALG- and PEG-coated, OVA-loaded chitosan nanoparticles were formulated using the ionotropic gelation method in a layer-by-layer fashion. This involved two stages: (a) For blank formulations, freeze-dried optimized chitosan-TPP nanoparticles were dispersed in ALG and PEG solutions to obtain ALG- and PEG-modified blank nanoparticles. (b) For the protein-loaded nanoparticles, OVA was added into chitosan solution before the addition of sodium tripolyphosphate (TPP). Then, the freeze-dried OVA-loaded chitosan nanoparticles were dispersed in the ALG and PEG solutions. The cationic chitosan nanoparticles prepared in this work had the capacity to adsorb anionic model protein (OVA) in aqueous solution via electrostatic interaction between them. Table 1 shows the effect of the OVA concentration on the chitosan nanoparticles’ size, zeta potential, and polydispersity index (PDI).

The average chitosan nanoparticles diameter increased after OVA loading in the range of 211 to 319 nm with increasing initial OVA concentration, while the PDI value also increased; however, the zeta potential values decreased. This could be attributed to the fact that negatively charged OVA was adsorbed onto the chitosan nanoparticles and neutralized some of the positive charges on the particles’ surfaces, thus decreasing the value of the zeta potential. The large nanoparticle sizes with high PDI and low zeta potential values are indicative of particle growth owing to lower amounts of polymer (0.2%) relative to the higher concentrations of protein (OVA) of 0.3% and 0.4%. The particle aggregation and subsequent increase in size and PDI create the potential for precipitation to occur, which results in the decreased dispersion of nanoparticles, homogeneity, and stability.

**Figure 1:**
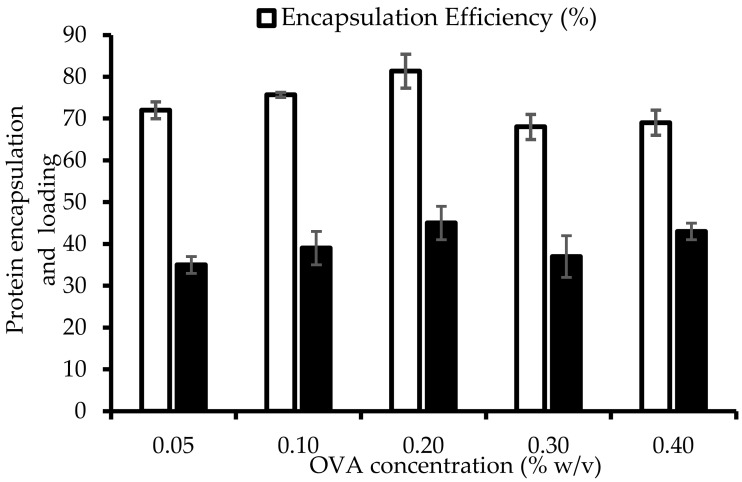
OVA encapsulation efficiency and loading capacity of chitosan nanoparticles (*n* = 3 ± SD).

At an initial OVA concentration of 0.05%, the encapsulation efficiency was 72% (Figure 1), which increased to 81% at a concentration of 0.2%.

However, at initial OVA concentrations of 0.3% and 0.4%, the protein encapsulation efficiency was reduced to 68% and 69%, respectively. This interesting observation might be attributed to the saturation from the absorption of OVA molecules on the chitosan nanoparticle surface at an OVA concentration of 0.2%, with the highest OVA encapsulation efficiency being 81% (Figure 1), after which the OVA encapsulation in chitosan nanoparticles was reduced. In this case, both the chitosan and OVA concentrations were 2 mg/mL, showing that formulations with an equal ratio (1:1) of host polymer (chitosan) and OVA protein had the highest encapsulation efficiency. Meanwhile, the zeta potential value also decreased from –17 to –13 mV at the OVA concentrations of 0.3% and 0.4%, respectively.

Higher zeta potential values in a dispersed system implies that the repulsive forces between particles is stronger, with reduced chances for aggregation; thus, the system is deemed to be moderately stable. In contrast, a lower zeta potential value implies reduced repulsive interaction between particles, resulting in a less stable system [21].

Therefore, based on the protein encapsulation efficiency, particle size, PDI, and zeta potential values, the 0.2% OVA-loaded chitosan nanoparticle formulation was considered optimized and was selected for ALG and PEG modification (coating).

#### 2.1.2. Effect of ALG Coating

The uptake of nanoparticles by intestinal PPs is greatly influenced by the size of nanoparticles and the surface properties. The desired particle size of nano complexes is necessary for improved oral protein-based vaccine delivery and particles below 1000 nm diameter are preferable for protein absorption through the intestinal epithelial membrane or PPs [22]. Chitosan is cationic in nature and can bind with mucin, and this can be enhanced further by the adhesive groups of a polymer such as ALG, ensuring a prolonged residence time on the intestinal mucosa layer and the subsequent improved absorption of adsorbed or encapsulated protein moieties [23]. The DLS profiles of OVA-loaded ALG-modified chitosan nanoparticles are shown in Table 2.

The particle size of the nanoparticles coated with ALG gels in concentrations from 0.05 to 0.4% ranged from 319 nm to 432 nm, which were higher than the sizes of the unmodified (Table 1) chitosan nanoparticles. This can be attributed to the ALG modified core shell present in the chitosan nanoparticles and the positive charge of the chitosan nanoparticles, which became negative (zeta potential value) after the ALG coating. Similar results were reported by Shukla et al.; [24], where blank chitosan particles exhibited positive zeta potential, but after being coated with ALG the zeta potential became negative. This might be because anionic ALG became more dominant over cationic chitosan on the nanoparticles’ surfaces. Interestingly, nanoparticles coated with 0.05% and 1.0% ALG showed a positive zeta potential for the OVA-loaded formulations. This may be explained by the fact that the surface charge of proteins derives mainly from the ionization of surface groups. Further, most proteins’ structures have hydrophobic nonpolar residues and hydrophilic polar groups, and their balance will affect the final surface charge [25]. The reduction in surface charge may be due to the exposure of non-polar hydrophobic residues within the protein secondary structure. Overall, these results indicate the augmentation of chitosan nanoparticles in the presence of ALG molecules due to the similar structural features of chitosan and ALG, causing electrostatic repulsion and forming a strong core shell on the surface of the nanoparticles.

#### 2.1.3. Effect of PEG Coating

PEG-coated blank chitosan nanoparticles showed sizes ranging from 262 to 282 nm, while the OVA-loaded particles ranged from 297 nm to 311 nm in size (Table 3). Increasing the PEG concentration from 0.05 to 0.4% caused the size of both the blank and OVA-loaded nanoparticles to increase (Table 3). Zhu and co-workers [26] reported that chitosan derivatives and PEG could form intermolecular hydrogen bonds between the electro-positive amino hydrogen of chitosan and the electro-negative oxygen atoms of PEG, forming colloidal carriers with a loose structure. After increasing the PEG concentration, the zeta potential value also increased gradually from −13 to −21 mV.

### 2.2. Analytical Characteristics

The FTIR, XRD, and DSC results can be seen in Supplementary Data, Sections S1 to S3 (Figures S1–S15).

### 2.3. Scanning Electron Microscopy (SEM)

**Figure 2:**
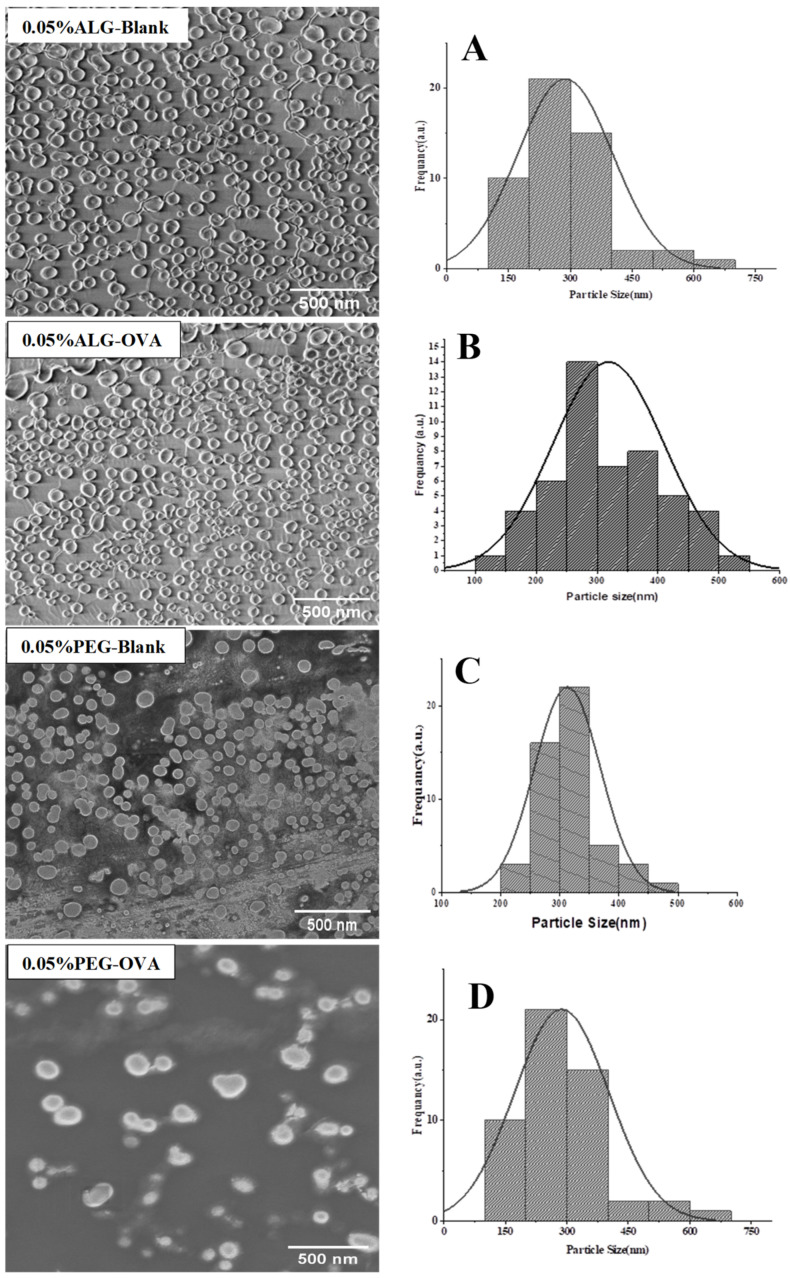
Representative SEM images of coated blank and OVA-loaded chitosan nanoparticles with corresponding size distribution histograms. The particle sizes in the SEM images are different to those in the corresponding graphs (histograms) because the latter were based on an estimate of selected particles over a particular field of view.

Figure 2 shows representative images of the nanoparticles coated with 0.05% ALG and PEG with corresponding size distribution profiles showing the effect of the protein incorporation. The OVA-loaded ALG-coated nanoparticles were spherical and sub-spherical. The diameter of both the blank and OVA-loaded nanoparticles showed sizes within the range of 2500–4500 nm, which was confirmed by DLS analysis. Figure S16 (Supplementary Data) shows that the chitosan nanoparticles coated with ALG at the lower concentrations (0.05–0.2%) exhibited uniform monodisperse and spherical shapes.

However, for ALG concentrations of 0.3% and 0.4%, larger sizes were observed for both blank and OVA-loaded nanoparticles. This is because the anionic nature of ALG allows binding with excess amounts of unreacted chitosan, thus forming bigger particles after coating. Furthermore, the OVA-loaded particles were larger in size than the blank ALG-coated nanoparticles. This could be because the negatively charged OVA was encapsulated within positively charged chitosan nanoparticles by electrostatic interaction, resulting in them having large sizes. The SEM images also suggest that nano-complexes are scattered independently over the colloidal solution, suggesting possible stabilization against self-aggregation. This result is consistent with a study reported previously by Li and co-workers, who showed that OVA-loaded cyclodextrin/chitosan nanoparticles used for oral delivery increased in size significantly from 213 to 836 nm [27].

On the other hand, the PEG-coated chitosan nanoparticles exhibited homogenous and spherical shapes and were compact in structure. Importantly, it was observed that increasing the PEG concentration did not affect the particle size, which was also confirmed by a DLS size measurement. This could be because, during the surface modification, PEG mainly interacts with the charged NH_2_ groups on the chitosan nanoparticle surface, with the corona of the nanoparticles comprising PEG and excess amounts of OVA. The size of the PEG-coated chitosan nanoparticles was observed to be in the 260–230 nm range, and nanoparticles coated with a PEG concentration of 0.4% were observed to have irregular shapes, as shown in Figure S16 (Supplementary Data). The PEG-coated blank nanoparticles were smooth, spherical, and uniformly dispersed, which was consistent for the PEG concentrations of 0.05 to 0.3%. This result is consistent with previously reported studies [28,29]. In these studies, it was shown that OVA-loaded PEG-modified chitosan nanoparticles had an average size from 193 nm to 200 nm, and the TEM image exhibited smooth spherical nanoparticles. In this project, the only difference observed was in the PEG molecular weight, with PEG-6000 (MW) being employed in our study, while Zhang and co employed PEG-1000 (MW).

### 2.4. Stability of Nanoparticles in Simulated Fluids

**Figure 3:**
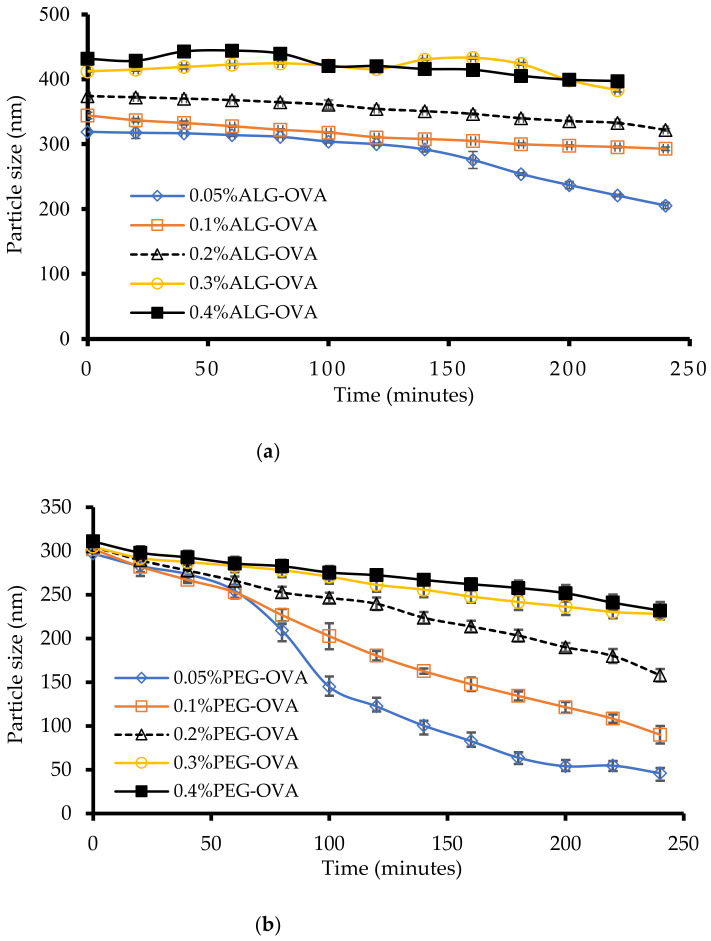
Simulated gastric fluid size stability analysis of (**a**) ALG-coated chitosan nanoparticles and (**b**) PEG-coated chitosan nanoparticles (*n* = 3 ± SD).

The most important role of nanoparticles in vaccine delivery is to protect protein molecules from the harsh GIT environment and transfer the encapsulated protein to the intestinal target site. Simulated gastric and intestinal fluid stability analyses of ALG- and PEG-coated chitosan nanoparticles were performed based on the size of the nanoparticles measured by DLS. The size of the ALG-coated chitosan nanoparticles was not significantly changed (*p* > 0.05) in the simulated gastric fluid (Figure 3a) compared with the PEG-coated chitosan nanoparticles (Figure 3b). The particle size distribution of the ALG-coated nanoparticles was multimodal (size fluctuations) at the higher ALG concentrations of 0.3% and 0.4%, as shown in Figure 3a. At the concentration of 0.05% ALG, the particle size was reduced in the gastric fluids, while at 0.1% and 0.2% ALG the particle sizes remained steady within a 4-hour time interval.

In comparing coated with unmodified chitosan nanoparticles, the latter showed no particles after 30 min in simulated gastric fluid. Therefore, it is evident that the ALG-coated chitosan nanoparticles remained stable in gastric fluid (as confirmed by the DLS size and SEM image) over a 4 h period and were able to encapsulate and potentially protect the protein from the gastric environment. On the other hand, the data on PEG-modified chitosan nanoparticles suggest that at the PEG concentrations of 0.05 and 0.1%, the size reduced significantly (*p* < 0.05) within 1 h, while for 0.3 and 0.4% PEG-coated chitosan nanoparticles, the size remained steady for 3 h (Figure 3b). In addition, a broad and multimodal size distribution was observed for the formulations coated with PEG at concentrations of 0.3 and 0.4%. This observation suggests that electrostatic repulsion between chitosan and PEG may have been weakened due to the change in the pH; therefore, the size of the particles was significantly reduced.

Another possible reason for this could be that the amino groups of the chitosan polymer could have been deprotonated and become negatively charged in the gastric fluid at lower PEG concentrations. Overall, these results suggest that the ALG- and PEG-coated chitosan nanoparticles maintained a negative charge and good stability in simulated gastric fluid, which is very important to allow the protein-loaded chitosan nanoparticles to reach the intestinal milieu, with particles containing an equal amount of (1:1) chitosan:ALG having the best stability in simulated gastric fluid. The results were further confirmed by observing the particles using scanning transmission electron microscopy (STEM), and the results are shown in Supplementary Files (Figures S17 and S18).

**Figure 4:**
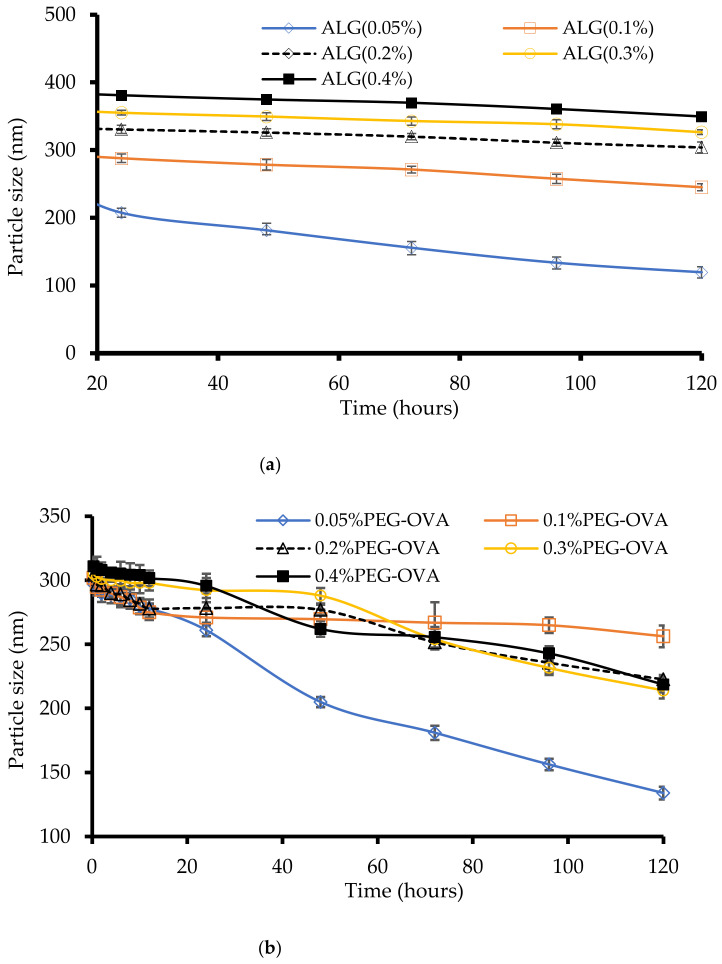
Simulated intestinal fluid analysis of (**a**) ALG-coated chitosan nanoparticles and (**b**) PEG-coated chitosan nanoparticles (*n* = 3 ± SD).

After passing through simulated intestinal fluid, the particle size was observed to remain steady over 120 h in ALG-coated chitosan nanoparticles (Figure 4a). Formulations coated with 0.05–0.2% of ALG showed monomodal size distribution peaks, and no significant reduction in particle size was observed for these three different ALG-coated chitosan nanoparticles. The zeta potential measurement of the nanoparticles showed that particles coated with 0.3 and 0.4% ALG were highly negatively charged with an increase in the zeta potential value. These high negative charges may have been due to the presence of anionic substances attached to the particle surfaces, such as phospholipids, bile salt, and peptides present in the intestinal fluid. Particle size and zeta potential measurements suggest that the particle size slowly reduced and that the slow degradation of chitosan nanoparticles occurred in the simulated intestinal fluid, which is supported by DLS and SEM (not shown) data. Overall, the chitosan nanoparticles coated with 0.3% ALG showed more stability against simulated intestinal fluid according to particle size analysis conducted using DLS and SEM. Moreover, subsequent in vitro protein release study also suggests that ALG-coated chitosan nanoparticles released protein in a sustained manner.

For the PEG-coated chitosan nanoparticles, formulations coated with 0.1% PEG exhibited relatively stable particle sizes in simulated intestinal fluid within 120 h (Figure 4b). On the other hand, the size of the chitosan nanoparticles coated with 0.05% PEG was significantly reduced (*p* < 0.05) within 120 h from 300 nm to 200 nm. A relatively low amount of PEG (0.05% and 0.1%) showed a significant reduction (*p* < 0.05) in the zeta potential values in these formulations. It is possible that the presence of bile salt and calcium ions in the medium react to remove PEG chains from the surface of the chitosan nanoparticles due to the low concentration of PEG or the corrosion of chitosan nanoparticles in intestinal medium through the degradation of the polymer chain.

Furthermore, the freeze-dried nanoparticle powder and suspension were stored at both room and fridge temperatures for a long-term stability evaluation. The nanoparticles’ size and zeta potential were almost the same over a 3-month period; however, at room temperature aggregation was observed within 10 days and size analysis by DLS or SEM showed bigger particles (data not shown).

### 2.5. In Vitro OVA Release from Nanoparticles

**Figure 5:**
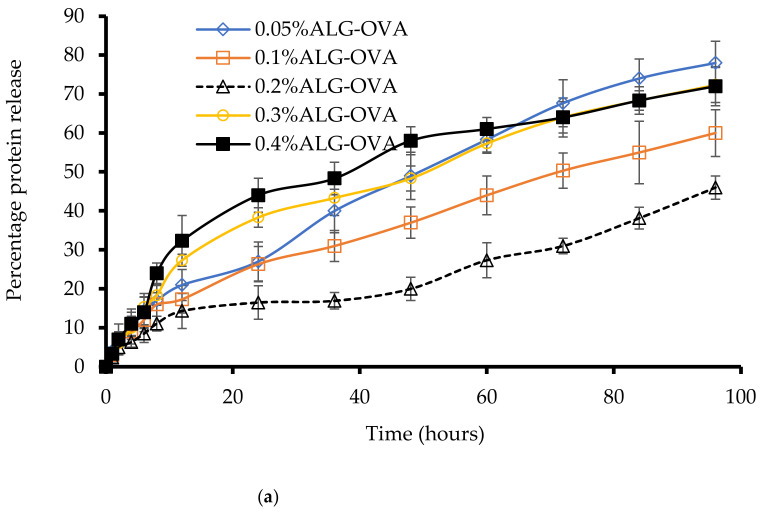

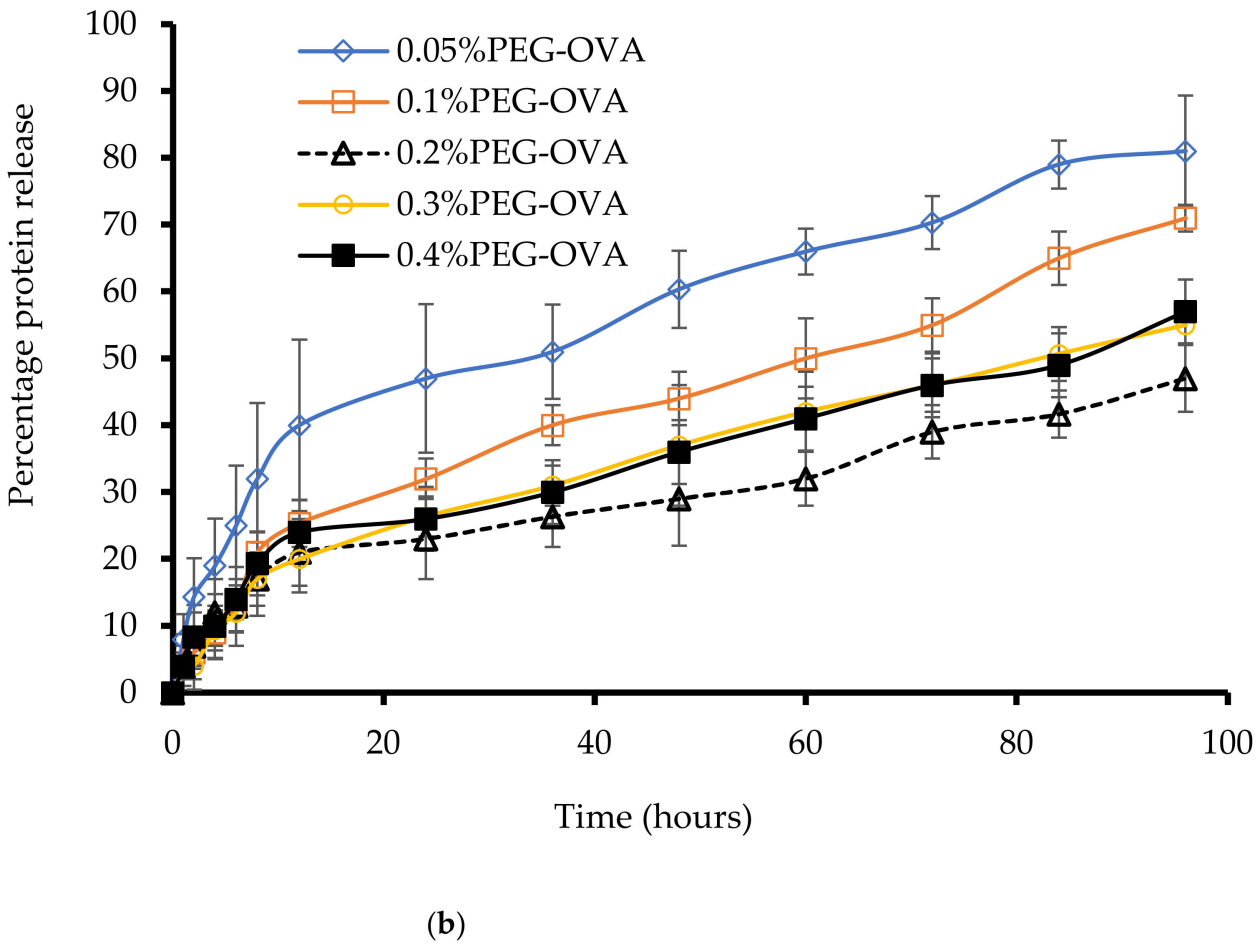
In vitro OVA release profiles from (**a**) ALG-coated chitosan nanoparticles and (**b**) PEG-coated nanoparticles in the presence of simulated intestinal fluid (*n* = 3 ± SD).

The cumulative amount of OVA released from the coated chitosan nanoparticles was determined in simulated intestinal fluid at pH 7.2 and the OVA concentration was estimated by measuring the absorbance via a Bradford assay. Three mechanisms played a key role in the release process: (i) normal diffusion through surface pores due to the concentration gradient, (ii) the charge screening of protein–nanoparticle interactions due to the presence of salts in simulated fluid, and (iii) the loss of electrostatic interaction via the protonation of carboxylate groups present in the nanoparticles [30]. The release profile of OVA from the ALG-coated chitosan nanoparticles is shown in Figure 5a. The ALG-coated nanoparticles demonstrated release in two stages, with rapid OVA release in the first 3 h followed by a gradual release for up to 96 h. The reason for the rapid initial release of OVA could be that the protein was loosely bound through electrostatic interactions in chitosan nanoparticles, which can easily be eroded in the ionic environment [31].

The second stage might correspond to protein molecules that were effectively encapsulated and tightly bound within the chitosan molecules. Further, ALG can increase the stability of nanoparticles’ structure, which leads to the sustained release of the OVA. For nanoparticles coated with 0.05% ALG, more than 78% of the OVA was released within 96 h, whereas particles coated with 0.1, 0.2, 0.3, and 0.4% ALG showed 60, 45, 72, and 72% OVA releases, respectively. Interestingly, particles coated with 0.3 and 0.4% ALG showed a higher protein release than 0.1 and 0.2%. This could possibly be due to the excessive amounts of ALG polymer present, which might be bound with the chitosan nanoparticles and form bigger particles; subsequently, the bile salt environment could have eroded the surface of these larger nanoparticles and invaded the internal structure. From Figure 5a, it can be observed that chitosan nanoparticles coated with 0.2% ALG could be more effective due to their slower and more sustained release of OVA, which is an important property for intestinally targeted protein-based mucosal vaccine delivery.

The OVA release profile from PEG-coated chitosan nanoparticles is shown in Figure 5b. A burst release was observed within 3 h, mainly due to the diffusion of protein from the particle surface. The particles coated with 0.05% PEG demonstrated an initial burst release of 25% of OVA within 3 h and an 81% release within 96 h. Particles coated with 0.1, 0.2, 0.3, and 0.4% PEG showed 71, 47, 55, and 60% of OVA releases, respectively, within 96 h. The particles coated with 0.2% PEG again showed a sustained and slow release of OVA, which could result from the diffusion of OVA through the polymer pores as well as the erosion and degradation of the polymer. This result confirms that chitosan nanoparticles with a steric PEG barrier could prevent the protein’s rapid release and prolong the presence of stable nanoparticles in a simulated intestinal environment.

The drug dissolution profiles do not show 100% protein release, as dissolution release was only performed over 96 h. This is because of the possibility of bacterial growth in dissolution medium beyond 96 h, which could affect the nanoparticle size and stability.

### 2.6. SDS–Polyacrylamide Gel Electrophoresis (SDS–PAGE) Analysis

**Figure 6:**
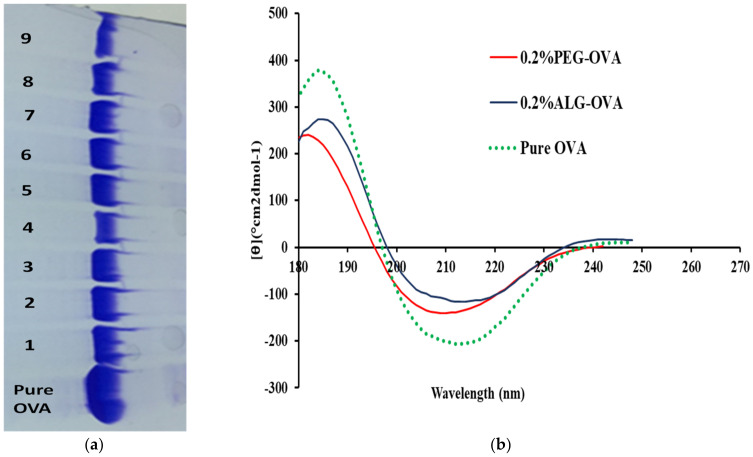
(**a**) SDS-PAGE analysis of OVA released from 0.2% PEG-coated chitosan nanoparticles (1–4) and from 0.2% ALG-coated chitosan nanoparticles (5–9) after 96 h compared to pure native OVA; (**b**) CD spectra of OVA protein after its release from ALG- and PEG-coated chitosan nanoparticles.

After the OVA release from the ALG- and PEG-coated nanoparticles, SDS-PAGE was performed to investigate any structural modification of the protein during release. After the respective recovery and re-suspension of OVA protein in phosphate buffer solution and OVA protein analysis by SDS-PAGE (Figure 6a), it was evident that a significant amount of protein was present after the release from the ALG-coated nanoparticles. Compared with the pure OVA protein band, it can be observed that the integrity of the OVA was maintained during nanoparticle formulation and upon release from the chitosan nanoparticles. The SDS-PAGE revealed that there were no additional bands; therefore, there was no indication of the presence of covalent aggregates or fragments besides the bands corresponding to monomeric OVA. Likewise, the profiles for the OVA released from chitosan nanoparticles coated with PEG was not significantly different to that of the ALG-coated particles, confirming the stability of the OVA during the protein release.

### 2.7. Circular Dichroism

The deformation of the main secondary structure (the α-helix) of the protein gives us a good idea of the protein distortion upon the exposure of the OVA-loaded nanoparticles. Figure 6b shows the CD spectra of OVA from representative ALG- and PEG-coated chitosan nanoparticles after 96 h in vitro release and diluted down to a final concentration of 120 µg/mL. It can be seen from the spectra that the minimum (208 nm) and maximum (220 nm) wavelength intensities were reduced but there were no major shifts, indicating that OVA’s secondary structure remained stable after exposure to simulated intestinal fluids during the in vitro release study. Table 4 shows the percentage of α-helicity and percentage of β- sheets of OVA after its release from ALG- and PEG-coated chitosan nanoparticles. Compared with pure OVA, the α-helical and β-sheet structure of OVA showed some distortion after release from the nanoparticles. The initial α-helicity of pure OVA was 23.1%, which decreased to 19.2% after a 96 h release study.

The reduction in α-helicity indicated that the nanoparticles were bound to the amino acid residues of the main peptide chain, breaking the hydrogen bonds, which reduced the α-helical content of the protein structure. This resulted in the unfolding of the consecutive polypeptides of OVA, which in turn exposed the hydrophobic regions of the proteins previously contained inside. However, the calculated values indicate that OVA can retain almost 84–86% of its initial secondary structure upon the exposure of the nanoparticles to intestinal fluid. Furthermore, it can also be seen from Table 4 that there was an increase in the % of β-sheets in the OVA released from 0.2% ALG-OVA at the concentration of 120 µg/mL compared to the β-sheet of pure OVA, confirming that these structural deformations were evidence of OVA being released from these nanoparticles after 96 h.

### 2.8. In Vitro Mucin Binding Mucoadhesion Assay

**Figure 7:**
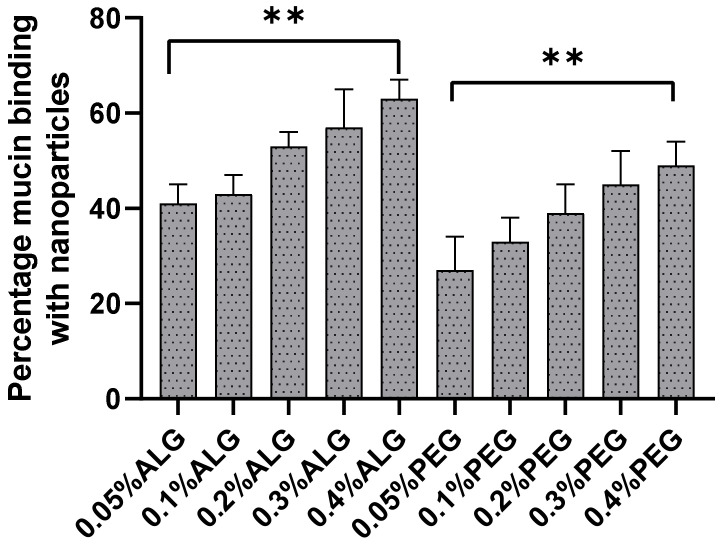
Mucin binding analysis of ALG- and PEG-coated chitosan nanoparticles (*n* = 3 ± SD) (** shows significant difference between the selected formulations).

In this study, the binding of mucin by the nanoparticles, which simulates how mucin associates with nanoparticles by self-assembly, was used to estimate the mucoadhesive behavior of the coated chitosan nanoparticles. Figure 7 shows that increasing the concentration of the ALG coating resulted in a higher level of mucin binding. For example, more than 63% of mucin was bound to the formulation coated with 0.4% ALG compared to the 41% bound by nanoparticles coated with 0.05% ALG, which was significantly different (*p* < 0.05). It can be observed from Figure 7 that generally, at higher concentrations of ALG, mucin binding was also higher. This is because of the positive charge of ALG-coated chitosan nanoparticles binding with negatively charged mucin. Moreover, chitosan also has mucoadhesive properties, as reported previously [32]. Therefore, ALG, which is a known mucoadhesive polymer, complements and further enhances the mucoadhesive characteristics of chitosan, as reported in other studies [33,34,35]. Moreover, at a lower pH, the ALG acid group is non-ionized and therefore exhibits a lower swelling ability. Hence, the ALG polymer directly binds with mucin through hydrogen bonds and increases the mucoadhesion. In this study, it is suggested that mucoadhesion is dependent on the properties of the nanoparticles’ surfaces and the amount of nanoparticles relative to those of mucin.

The excellent mucoadhesive properties of the ALG-coated chitosan nanoparticles could make them useful as a novel particulate system to develop a mucoadhesive vaccine delivery system. As depicted in Figure 7, increasing the concentration of PEG also increased the mucin binding efficiency. This may be due to the complex structure of the nanoparticles’ surfaces and the density of the nanoparticles when dispersed in solution. Furthermore, the particles will swell and form strong bonds with the mucin due to the negative charge of mucin and the positive charge of nanoparticles. Chitosan nanoparticles coated with 0.4% PEG showed 48% mucin binding, while those coated with 0.05% PEG showed 28% mucin binding, which is lower than that for both ALG-coated and the uncoated (data not shown) chitosan nanoparticles. PEG-coated chitosan nanoparticles added into mucin solution at simulated intestinal fluid pH probably detached from the mucin because of rapid swelling.

### 2.9. Ex Vivo Mucoadhesion Test

**Figure 8:**
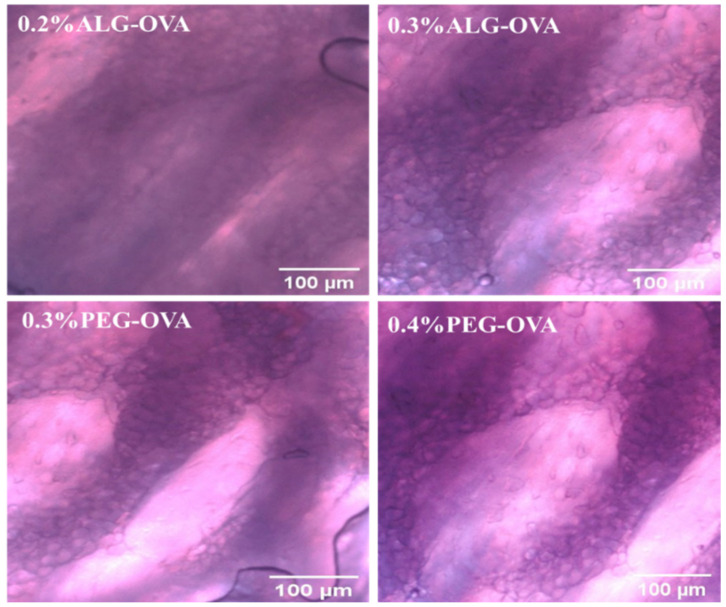
Fluorescence microscopy images showing the mucoadhesion of unlabeled ALG- and PEG-coated chitosan nanoparticles (control) to the pig oral mucosa. Extracted images were analyzed using the Image J software in red to green (R/G) intensity and no green fluorescence was observed as a distinguishing feature for the samples compared with Figure 9 below.

**Figure 9:**
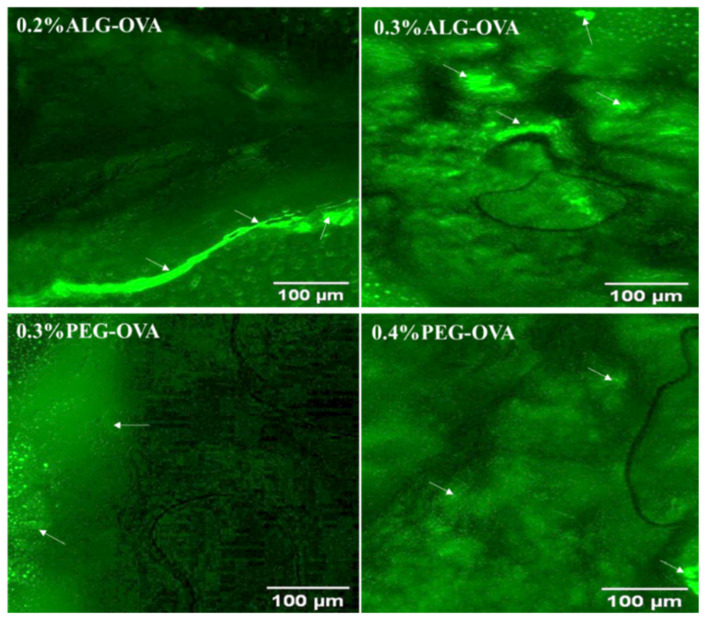
Fluorescence microscopy image showing the mucoadhesion of FITC-labelled ALG- and PEG-coated chitosan nanoparticles to the pig oral mucosa (white arrows show areas of intense green fluorescence on the mucosa where nanoparticles were resident). The fluorescence images were analyzed to extract the green intensities using the Image J software. This was used to determine nanoparticle presence (arrow bar) on the pig mucosa tissue. The images obtained for 0.2% ALG-OVA nanoparticles showed a progressive increase in the intensity of green fluorescence emitted from the FITC-labelled particles of this formulation on pig mucosa tissue compared to the 0.3% ALG-OVA. This indicates that the 0.2% ALG-coated particles could potentially be more adhesive and possess a higher binding efficacy than the 0.3% ALG-coated particles. In the case of the PEG-coated samples, the 0.3% PEG-modified chitosan nanoparticles showed higher green fluorescence intensities and therefore better adhesion than the 0.4% PEG-modified nanoparticles.

Ex vivo mucoadhesion studies were performed with pig oral mucosa using coated and OVA-loaded chitosan nanoparticles to further investigate the mucoadhesion properties of the nanoparticles. Based on the mucin binding results and the particle size properties from earlier sections, four optimized coated formulations were selected. The quantity of nanoparticles taken up by tissues was determined indirectly by measuring the quantity of free nanoparticles using a fluorescence plate reader. All the studies were performed by maintaining a mucus layer on the top side without washing it off, so that result could reflect the real-life environment. Figure 8 shows the fluorescence tissue image for ALG- and PEG-coated nanoparticles with no FITC labelling (control), while Figure 9 depicts the fluorescence images of tissues, with FITC-labeled ALG- and PEG-coated nanoparticles showing fluorescence after 2 h of contact.

From the initial 100 µg loading, the oral pig mucosal tissues took up approximately 45 µg within 60 min for the ALG (0.2%) -coated nanoparticles and 49 µg for the ALG (0.3%) -coated chitosan nanoparticles. On the other hand, the PEG-coated chitosan nanoparticles showed 21 µg and 25 µg uptakes for 0.3 and 0.4% PEG, respectively. The chitosan nanoparticles coated with 0.3% ALG showed the maximum mucoadhesive value in the ex vivo analysis, which was significantly (*p* < 0.05) higher than that of both PEG-coated nanoparticle formulations and confirmed the results of the mucin interaction study results described in Section 2.8. This shows that ALG-coated chitosan nanoparticles have a higher potential as a mucosal vaccine system to deliver protein-based antigen for mucosal immunity.

Although the oral mucosa does not fully simulate the main target region, which is the PPs within the small intestines, it was a suitable mucin-bearing mucosa model with a very strong intact mucus barrier layer and gave an indication of the nanoparticles’ ability to remain on a target mucosa site. Furthermore, it was easier and quicker to retrieve the oral mucosa from the pig slaughterhouse and process it more quickly with minimal cleaning requirements, unlike the small intestines. However, this is a limitation, and future studies involving the exact target intestinal mucosa site will be required. Overall, the results show that there is potential for the attached nanoparticles to form a temporary depot system for the gradual continuous activation and recruitment of antigen-presenting cells (APC).

## 3. Materials and Methods

### 3.1. Materials

Chitosan (medium molecular weight: 75–85% deacetylated), sodium tripolyphosphate, ovalbumin, and 96% electrophoresis agarose gel were purchased from Sigma-Aldrich, (Gillingham, UK). Mucin from porcine stomach, glacial acetic acid, calcium chloride, Coomassie brilliant blue, disodium hydrogen phosphate, hydrochloric acid, monobasic potassium dihydrogen phosphate, sodium acetate, sodium azide, sodium bicarbonate, sodium chloride, sodium dihydrogen phosphate, sodium hydroxide, D(+)-trehalose, polyethylene glycol (MW 20,000), Bradford protein assay reagent, pepsin from porcine gastric mucosa, sodium taurocholate, lecithin, maleic acid, sodium oleate, sodium alginate- medium viscosity, SKU: A2033 from brown algae, porcine bile extract, ammonium nitrate, potassium chloride, potassium citrate, urea, uric acid sodium salt, glycine, lactic acid sodium salt, 1 mM ethylenediamine tetraacetic acid (EDTA), 2-mercaptoethanol, and bromophenol blue sodium dodecyl sulfate-acrylamide gel electrophoresis (SDS-PAGE) were all purchased from Bio-Rad, Watford, UK.

### 3.2. Preparation of Chitosan Nanoparticles

Chitosan nanoparticles were prepared by following the ionotropic gelation method, as previously reported for optimized chitosan-tripolyphosphate blank nanoparticles [36], using medium-molecular-weight chitosan. Briefly, 2 mg/mL of chitosan was prepared by dispersing the polymer powder in aqueous acetic acid (1% v/v) and stirred continuously (60 min) until it was completely dissolved. Then, 1 mg/mL of sodium tripolyphosphate (TPP) in deionized water was added dropwise to the chitosan solution with mechanical stirring (600 rpm). The nanoparticles of chitosan–TPP suspension were formed during stirring at room temperature for 60 min, and the sample was collected as blank nanoparticles for characterization. Quantitative characterization experiments were performed in triplicate and data are shown as mean values ± standard deviations.

### 3.3. Loading of OVA into Chitosan Nanoparticles

The OVA-loaded chitosan nanoparticles were prepared in a similar fashion as that described in Section 3.2 above. OVA was added dropwise into chitosan solution in aqueous acetic acid during stirring and TPP suspension was added (for nanoparticle preparation) as described above and sonicated for 2.5 min by ultra-sonication (Benchtop 20 L, Medisafe, UK Ltd., Bishop’s Stortford, UK) to disaggregate the nanoparticles. The encapsulation procedure was performed at different concentrations (0.05, 0.1, 0.2, 0.3, and 0.4%) of OVA with chitosan nanoparticles under mild agitation for 5 h at room temperature. The encapsulation efficiency and loading capacity of OVA onto chitosan nanoparticles were detected in an indirect way by determining the free OVA remaining in the supernatant after centrifugation. Briefly, 1 mL of OVA-loaded chitosan nanoparticle suspension was centrifuged (Hitachi, Chiyoda City, Tokyo, Japan) at 20,000 rpm for 20 min and the concentration of OVA in the supernatant was measured using the Bradford protein assay kit (Bio-Rad, Watford, UK). The standard curve was prepared according to the company protein assay manual. The supernatant of blank chitosan nanoparticles was adopted as the blank to correct the absorbance reading value of the OVA-loaded chitosan nanoparticles. The corrected optical density (OD) value was then used to calculate the concentration of OVA in the supernatant.

The encapsulation efficiency and loading capacity values were calculated using Equations (1) and (2):

(1) $$\mathrm{Encapsulation}\;\mathrm{efficiency}\left(\%\right)=\frac{\mathrm{total}\;\mathrm{amount}\;\mathrm{of}\;\mathrm{OVA}-\mathrm{free}\;\mathrm{OVA}\;\mathrm{in}\;\mathrm{supernatent}\;}{\mathrm{total}\;\mathrm{amount}\;\mathrm{of}\;\mathrm{OVA}}\times 100$$

(2) $$\mathrm{Loading}\;\mathrm{capacity}\left(\%\right)=\frac{\mathrm{total}\;\mathrm{amount}\;\mathrm{of}\;\mathrm{OVA}-\mathrm{free}\;\mathrm{OVA}\;\mathrm{in}\;\mathrm{supernatent}}{\mathrm{Dried}\;\mathrm{nanoparticles}\;\mathrm{weight}\;}\times 100$$

### 3.4. Preparation of ALG- and PEG-Coated Chitosan Nanoparticles

The blank and OVA-loaded nanoparticles prepared above were coated with ALG and PEG. The freeze-dried nanoparticles were dispersed in aqueous buffer solution with a pH of 7.2 and added dropwise into the ALG solution (pH 7.2) at different concentrations (0.05, 0.1, 0.2, 0.3, and 0.4% w/v) under mild agitation for 20 min. The resulting suspension was centrifuged at 20,000 rpm for 30 min and the supernatant was discarded. Finally, ALG-coated chitosan nanoparticles were re-dispersed into 0.524 mmol/L aqueous calcium chloride solution (pH 7.2) to crosslink the ALG layer present on the surface of the chitosan particles. The same procedure was followed to prepare PEG-coated chitosan nanoparticles with different concentrations of PEG (0.05, 0.1, 0.2, 0.3, and 0.4% w/v) but with no crosslinking step.

### 3.5. Analytical Characterization

The particle size, polydispersity index (PDI), and zeta potential were analyzed by dynamic light scattering (DLS) with the help of a Zetasizer Nano-ZS90 (Malvern Instruments, Worcester, UK) using a disposable sizing cuvette for size and PDI analyses. Zeta potential was measured using a reusable folded capillary zeta cell (Malvern Model: DTS1070). The sample was measured in double-distilled water and adjusted to a conductivity of 50 IS/cm with sodium chloride solution (0.9% w/v). The pH was in the range of 5.5–7.5 and the applied field strength was 20 V/cm. The sample was properly diluted with an appropriate dilution medium and scanned with constant refractive index viscosity and dielectric constant for all formulations. The measurements were carried out at a position of 4.65 mm from the cuvette wall with an automatic attenuator and at a controlled temperature of 20 °C. For each sample, 15 runs of 10 s were performed in triplicate (*n* = 3) and data were presented as mean ± SD.

Physico-chemical properties were analyzed by Fourier transform infrared (FTIR) spectroscopy, X-ray diffraction (XRD), and differential scanning calorimetry (DSC), details of which can be found in the Supplementary Data (Sections S1–S3).

### 3.6. Scanning Electron Microscopy (SEM)

The morphology of the nanoparticles was examined by SEM (Hitachi SU8030) (Chiyoda City, Tokyo, Japan) at an operating voltage of 1 kV. One drop of freshly prepared particle suspension was deposited onto the sample stub and left to dry in air and the dried sample was coated with chromium. For transmission electron microscopy analysis, a 30 kV high-resolution scanning transmission electron microscope (STEM)(Hitachi SU8030, Chiyoda City, Tokyo, Japan ) was used to examine the morphological characteristics of the OVA-loaded nanoparticles. One drop of sample suspension was placed on a 300 mesh copper grid and allowed to sit on the grid for 10 min until it air dried. Excess liquid was removed by using filter paper before observation on the STEM machine.

### 3.7. Preparation of Simulated Fluids

The simulated gastric fluid was prepared in double-distilled water by the addition of 1.594 g/L sodium chloride, 0.328 g/L ammonium nitrate), 0.636 g/L potassium phosphate, 0.202 g/L potassium chloride, 0.308 g/L potassium citrate, 0.021 g/L uric acid sodium salt, 0.198 g/L urea, 0.146 g/L lactic acid sodium salt, and 3% *w*/*w* porcine stomach mucin. The pH was then finally adjusted to 1.2 using 37% (*v*/*v*) hydrochloric acid. The simulated intestinal fluid was prepared by the addition of 54 g/L bile salt solution and 24 g/L pancreatic lipase with no pepsin added, before finally adjusting the pH to 7.2.

### 3.8. Stability of Nanoparticles in Simulated Fluids

The stability of the nanoparticles was analyzed by dispersing the freeze-dried coated and protein-loaded chitosan nanoparticles in simulated gastric fluid (pH 1.2) and simulated intestinal fluid (pH 7.2) at room temperature (20 °C), in a water shaking bath (150 rpm), and samples were withdrawn at predetermined time intervals. However, to determine the long-term stability of the nanoparticles, both the suspension and powder forms were maintained over 3 months using sodium azide (suspension state) as a preservative and stored at 4 °C. The size, zeta potential, and SEM/STEM morphology were then measured to confirm the stability of the nanoparticles in both fluids.

### 3.9. In Vitro Protein Release Study

To determine the amount of protein released from the chitosan nanoparticles, the Bradford reagent (protein-specific dye, Coomassie brilliant blue) was used and analyzed in a 96-well polystyrene plate. Freeze-dried protein-loaded nanoparticles (5 mg) were dispersed in 0.01% sodium azide containing 2 mL of PBS (pH 7.2), simulating intestinal fluid, and incubated at 37 °C in bath shaker with gentle stirring. At pre-determined time intervals of 0, 1, 2, 4, 6, 8, 10, 12, 24, 36, 48, 60, 72, and 96 h, 1.0 mL aliquots of the dissolution medium were collected and replaced with the same volume of fresh PBS. The samples were centrifuged at 15000 rpm and nanoparticle pellet was separated from the supernatant and discarded. The supernatant was analyzed for OVA in triplicate (*n* = 3) with an absorbance plate reader at a wavelength of 595 nm and the Bradford reagent standards were used according to the manufacturer’s instructions for the kit.

### 3.10. SDS–Polyacrylamide Gel Electrophoresis (SDS–PAGE)

SDS-PAGE was performed on OVA released from surface-modified nanoparticles to determine the primary structure after 96 h in simulated intestinal fluid (pH 6.8). Aliquots (20 μL) of these dispersions were mixed with a similar volume of SDS loading buffer in a 1 mL centrifuge tube and the mixture was heated to 80 °C for 10 min to denature the proteins. After 10 min, the tube was cooled to room temperature and centrifuged to remove any suspended solids. Then, 15 μL aliquots of the supernatant was transferred to the polyacrylamide gradient gel and electrophoresis was performed at 180 V over 120 min [37]. The loading buffer comprised tris-HCl, pH 6.8, 1 mM ethylenediamine tetra acetic acid (EDTA), 2-mercaptoethanol, and bromophenol blue served as the tracking agent. 2-mercaptoethanol was the reducing agent, and it was responsible for breaking the disulfide bridges and denaturing the protein molecules. The gel was stained with Coomassie blue staining solution and washed with 5% (v/v) acetic acid solution overnight, and images were taken using an UV-visible imaging (Geldoc) system(Bio-Rad, Watford, UK).

### 3.11. Circular Dichroism

Circular dichroism (CD) spectra were obtained from a Chirascan spectrophotometer (Applied Photophysics Limited (Leatherhead, UK) to examine the secondary structure of OVA during the release period. OVA was extracted from samples after a 96 h in vitro release study by centrifuging at 5000 rpm before the CD analysis and compared with native OVA. Spectra were collected at 20 °C using a quartz cell (path length 0.05 cm) in a wavelength range of 180 to 260 nm, with a resolution of 0.2 nm and a 2.25 s response time. Each spectrum represented an average of four consecutive scans. Noise reduction, blank solution subtraction, and data analyses were performed using standard analysis and a temperature/wavelength analysis program (Origin pro 8, Northampton, MA, USA). The CD results expressed in terms of mean residues ellipticity (MRE) can be denoted by Equation (3).

(3) $$\mathrm{MRE}=\frac{\mathrm{CD}\;\left(\mathrm{obs}\right)}{\mathrm{CpNI}}\times 10\;$$

where observed CD is measured in mol deg, Cp is the molar concentration of OVA, N is the number of amino acid residues (385 for OVA), I is the path length, and MRE is in units of cm^2^ mol^−1^.

MRE can be used to calculate the α-helix content of OVA using Equation (4):

(4) $$\alpha -\;\mathrm{helix}\;(\%)=\frac{-\left[\theta \right]208-4000}{33000-4000}$$

where $\left[\theta \right]208$ is the observed MRE value at 208 nm, 4000 is the MRE of the β-form and random coil conformation cross at 208 nm, and 33,000 is the MRE value of a pure α- helix at 208 nm.

### 3.12. In Vitro Mucin Binding Mucoadhesion Assay

The mucoadhesive properties were investigated as previously described [38] but with minor modifications. Briefly, 0.5 mL of mucin solution (0.5 mg/mL) was mixed with 0.5 mL of each surface-modified nanoparticle suspension and incubated at 37 °C in a shaking water bath for 2 h. After centrifugation at 16000 rpm for 40 min, the supernatant was collected and the amount of free mucin was measured using the Bradford protein assay. The supernatant was incubated with Bradford reagent for 5 min in a 96-well plate, after which the absorbance (620 nm) was measured with an auto absorbance reader (Multiskan FC Microplate Photometer, Thermo Fisher Scientific, (Loughborough, UK). The mucin concentration was calculated from a standard curve with concentrations ranging from 0.1 to 1.0 mg/mL.

### 3.13. Ex Vivo Mucoadhesion Study

Pig maxilla (from 5 months old pig, weighing around 75–80 kg) was obtained from a local slaughterhouse (East Sussex, Rotherfield, UK) and transported to the laboratory in ice-cold isotonic phosphate buffer (pH 7.4). This process took no longer than 45 min. The tissue was prepared according to the methodology previously described [39], with minor modifications. Within 2 h of slaughter, samples of palatal, gingival, tongue (from the upper two-thirds section), and buccal (from the cheek region) mucosa were separated from the underlining tissue using a scalpel and rinsed with saline. Mucosa with any visual surface damage was discarded. Intact mucosa was immersed in deionized water at 65 °C for 60 s, and the epithelium was carefully separated from the connective tissue. It was reported previously that this procedure did not influence the epithelium integrity of the esophageal and buccal mucosa [40]. The experiments were conducted using fluorescein isothiocyanate (FITC)-labeled ALG- and PEG-coated OVA-loaded chitosan nanoparticles to enable observation and imaging under a fluorescence microscope (Olympus IX70 microscope fitted with a CCD camera, FITC, TxR and Nomarski optics, (Shinjuku City, Tokyo, Japan ), with the unlabeled equivalents sued as controls. All the experiments were conducted with mucosa from at least three different animals (*n* = 3).

### 3.14. Statistical Analysis

All the quantitative data were analyzed by one-way ANOVA using the GraphPad Prism software (version 5.02) (San Diego, CA, USA). The level of significance was set at *p* < 0.05.

## 4. Conclusions

The main purpose of this study was to determine chitosan nanoparticles’ stability in simulated fluids and investigate the mucoadhesive properties for the potential delivery of protein antigen via the GIT to potentially elicit a mucosal immune response. As an important finding, it was demonstrated that the ALG-modified chitosan nanoparticles showed more stability than PEG coated nanoparticles in simulated gastric and intestinal fluid and sustained protein release properties. It was also demonstrated that the particle’s surface charge, hydrophobicity, size and zeta potential can influence their stability after coating. The OVA present in the ALG-coated chitosan nanoparticles did not undergo significant structural changes after 96 h of release study, which was confirmed by SDS-PAGE and CD. The ALG-coated nanoparticles also showed better mucin binding and ex vivo mucosa mucoadhesion than the PEG-coated equivalent. Given these properties, chitosan-ALG nanoparticles have the potential to be developed as a protein antigen carrier for oral vaccine delivery to achieve mucosal immunity.

## Figures and Tables

**Table 1:** Effect of different initial concentrations of OVA in chitosan-TPP nanoparticles on particle size, PDI, and zeta potential. Each value is the mean of three independent measurements (*n* = 3 ± SD).

Initial OVA Concentration (% *w/v*)	Particle Size (± SD) (nm)	Zeta Potential (± SD) (mV)	PDI (± SD)
0.05	211 ± 5	12 ± 1	0.26 ± 0.01
0.10	234 ± 2	15 ± 1	0.29 ± 0.00
0.20	254 ± 1	–21 ± 0	0.25 ± 0.01
0.30	287 ± 6	–17 ± 2	0.34 ± 0.00
0.40	319 ± 5	–13 ± 1	0.38 ± 0.02

**Table 2:** Effect of anionic ALG concentration on chitosan nanoparticles after coating. The optimized nanoparticles were prepared from 0.2% chitosan solution whilst the OVA initial concentration was 0.2% (1:1 ratio) (*n* = 3 ± SD).

ALG Concentration (% *w/v*)	Particle Size ± SD (nm)		Zeta Potential Value ± SD (mV)		PDI (±SD)	
	OVA-Loaded	Blank	OVA-Loaded	Blank	OVA-Loaded	Blank
0.05	319 ± 3	275 ± 5	21 ± 5	9 ± 1	0.23 ± 0.06	0.19 ± 0.01
0.10	345 ± 8	288 ± 5	21 ± 7	–13 ± 0	0.25 ± 0.03	0.21 ± 0.00
0.20	375 ± 5	316 ± 3	–24 ± 3	–14 ± 1	0.29 ± 0.01	0.25 ± 0.00
0.30	412 ± 6	356 ± 4	–26 ± 1	–16 ± 1	0.32 ± 0.07	0.27 ± 0.01
0.40	432 ± 10	377 ± 6	–29 ± 0.4	–19 ± 1	0.36 ± 0.05	0.29 ± 0.00

**Table 3:** Effect of PEG coating on the size, zeta potential, and PDI of chitosan nanoparticles. The optimized nanoparticles were prepared from 0.2% chitosan solution whilst the OVA initial concentration was 0.2% (1:1 ratio) (*n* = 3 ± SD).

PEG Concentration (% *w/v*)	Particle Size ±SD (nm)		Zeta Potential ± SD (mV)		PDI (± SD)	
	OVA-Loaded	Blank	OVA-Loaded	Blank	OVA-Loaded	Blank
0.05	297 ± 6	262 ± 5	–13 ± 1	9 ± 2	0.21 ± 0.04	0.20 ± 0.01
0.10	302 ± 9	276 ± 7	–16 ± 1	9 ± 1	0.22 ± 0.01	0.20 ± 0.0
0.20	305 ± 4	279 ± 4	–18 ± 0.9	13 ± 2	0.25 ± 0.03	0.24 ± 0.01
0.30	305 ± 4	282 ± 5	–20 ± 2	14 ± 1	0.31 ± 0.01	0.25 ± 0.01
0.40	311 ± 7	286 ± 9	–20 ± 1	17 ± 3	0.3 ± 0.05	0.28 ± 0.01

**Table 4:** Calculated % of α-helicity and % of β-sheets of OVA after release from ALG- and PEG-coated chitosan nanoparticles.

Sample Name	α-Helix (%)	β-Sheet (%)	Random Coil (%)
Native OVA	23.1	17.5	56.0
0.2% ALG-OVA	21.1	19.5	53.0
0.2% PEG-OVA	29.4	16.3	52.0

## Data Availability

Not applicable.

## Supplementary Materials

The following supporting information can be downloaded at https://www.mdpi.com/article/10.3390/md20030156/s1. Figure S1: FTIR spectra of pure starting materials; Figure S2: FTIR analysis of ALG-coated blank chitosan nanoparticles; Figure S3: FTIR spectra of ALG-coated OVA-loaded chitosan nanoparticles; Figure S4: FTIR spectra of PEG-coated blank chitosan nanoparticles; Figure S5: FTIR spectra of PEG-coated OVA-loaded nanoparticles; Figure S6: XRD diffractograms of pure materials; Figure S7: X-ray diffractograms of ALG-coated blank chitosan nanoparticles; Figure S8: X-ray diffractograms of ALG-coated OVA-loaded chitosan nanoparticles; Figure S9: X-ray diffractograms of PEG-coated blank chitosan nanoparticles; Figure S10: X-ray diffractograms of PEG-coated OVA-loaded chitosan nanoparticles; Figure S11: DSC thermograms of pure materials; Figure S12: DSC thermograms of ALG-coated blank nanoparticles; Figure S13: DSC thermograms of OVA-loaded alginate-coated chitosan nanoparticles; Figure S14: DSC thermograms of PEG-coated blank chitosan nanoparticles; Figure S15: DSC thermograms of OVA-loaded PEG-coated chitosan nanoparticles; Figure S16: SEM images of blank and OVA-loaded ALG and PEG-coated chitosan nanoparticles with different concentrations of ALG/PEG coating; Figure S17: S(a) representative STEM image of 0.2%ALG-OVA in simulated gastric fluid after 240 minutes (b) representative STEM image of 0.2%PEG-OVA in simulated gastric fluid after 240 minutes.; Figure S18: (a) STEM images 0.2%ALG-OVA, sample analyzed after 120 hours in simulated intestinal fluid (b) STEM images of 0.2%PEG-OVA, sample analyzed after 120 hours in simulated intestinal fluid.
