# Supplementary material for: Enhancing Stability and Mucoadhesive Properties of Chitosan Nanoparticles by Surface Modification with Sodium Alginate and Polyethylene Glycol for Potential Oral Mucosa Vaccine Delivery
Source: Mar Drugs. 2022 Feb 22;20(3):156. doi: 10.3390/md20030156 (PMC8953124; doi:10.3390/md20030156)
Supplement: Supplementary file 1 [file marinedrugs-20-00156-s001.zip › marinedrugs-1591000-supplementary.pdf]

marine drugs

MDPI

# Supplementary Information

# Enhancing Stability and Mucoadhesive Properties of Chitosan Nanoparticles by Surface Modification with Sodium Alginate and Polyethylene Glycol for Potential Oral Mucosa Vaccine Delivery

**Muhammad Khairul Amin and Joshua Siaw Boateng***

School of Science, Faculty of Engineering and Science, University of Greenwich at Medway, Central Avenue, Chatham Maritime, Chatham, Kent ME4 4TB, United Kingdom; khairulsub@gmail.com
* Correspondence: j.s.boateng@gre.ac.uk; joshboat40@gmail.com

## Results

### S1 Fourier Transform Infrared (FTIR) analysis

FTIR spectroscopy was employed for investigating the formation and coating of chitosan nanoparticles and protein-polymer interaction in formulation and the results are shown in figures S1 – S5. The FTIR spectrum of pure materials (figure S1) showed a strong absorption band at 3446.79 $cm^{-1}$ which would signal the presence of OH group in ALG and NH stretching vibration. The broader peak indicates that hydrogen bonding has been enhanced the pure materials (chitosan and OVA). The pyranose ring is indicated by the asymmetric and symmetric $CH_2$ stretching vibration at 2424 and 2825 $cm^{-1}$. The peak around 1638-1641 $cm^{-1}$ is due to C- O asymmetric stretching, the peak at 1458 $cm^{-1}$ is the C -O symmetric stretching in blank nanoparticles, $CH_2$ bending and OH deformation and the peak at 1271$cm^{-1}$ indicate P- O stretching. The peaks at 1320, 1303, 1157 and 1070 $cm^{-1}$ indicate the presence of C-O stretching, OH in plane blending, and C-O-C linkage respectively in 0.3% ALG coated formulation. The peak at 1020 $cm^{-1}$ was for P-O, C-OH stretching C-N vibrations and peaks at 877 $cm^{-1}$ were due to N-H deformation in OVA loaded formulation. The peaks at 769 and 534.28 $cm^{-1}$ were indicative of N-H wagging and OH out of plane bending vibrations respectively.

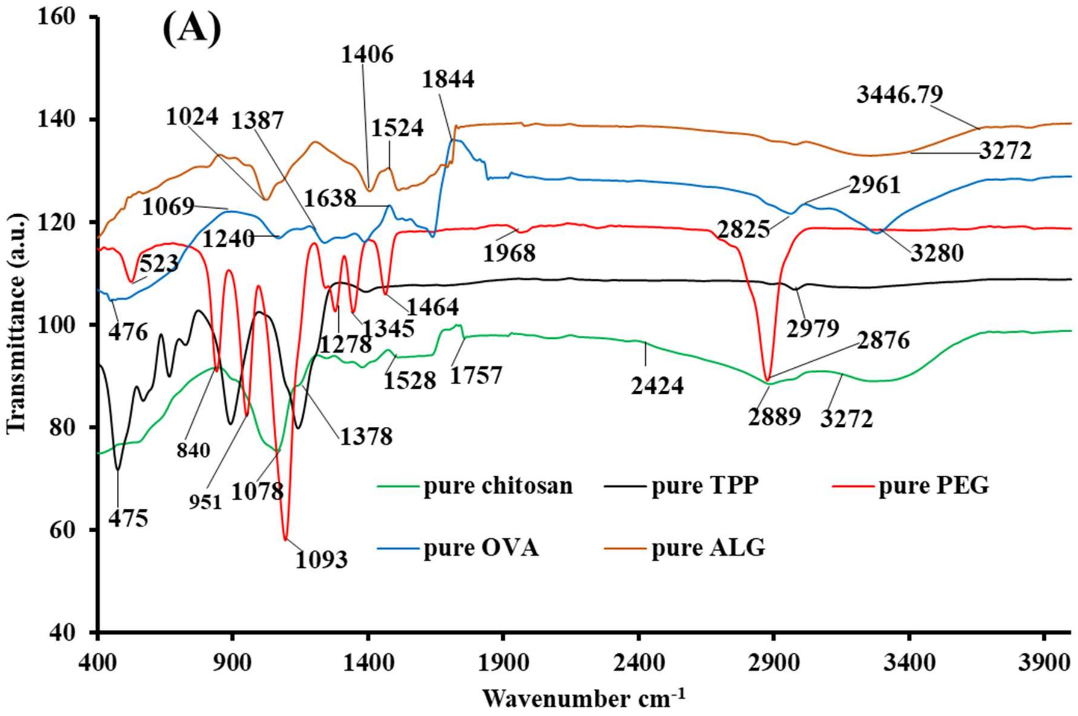


**Figure S1.** FTIR spectra of pure starting materials.

According to figure S2, the ALG coated blank chitosan nanoparticles showed a similar spectrum to the OVA loaded ALG coated chitosan nanoparticles in figure S3. The peaks at 3435, 1640, 1415 and 1109 $cm^{-1}$ were due to the stretching of O-H, COO- (asymmetric), COO (symmetric) and C-O-C, respectively. Comparing ALG coated blank and OVA loaded formulation, there was a shift of COO peaks at 1615 and 1415 $cm^{-1}$ to higher wavenumbers (1640 and 1418 $cm^{-1}$) respectively, as well as a decrease in intensities for the OVA loaded ALG coated nanoparticles.

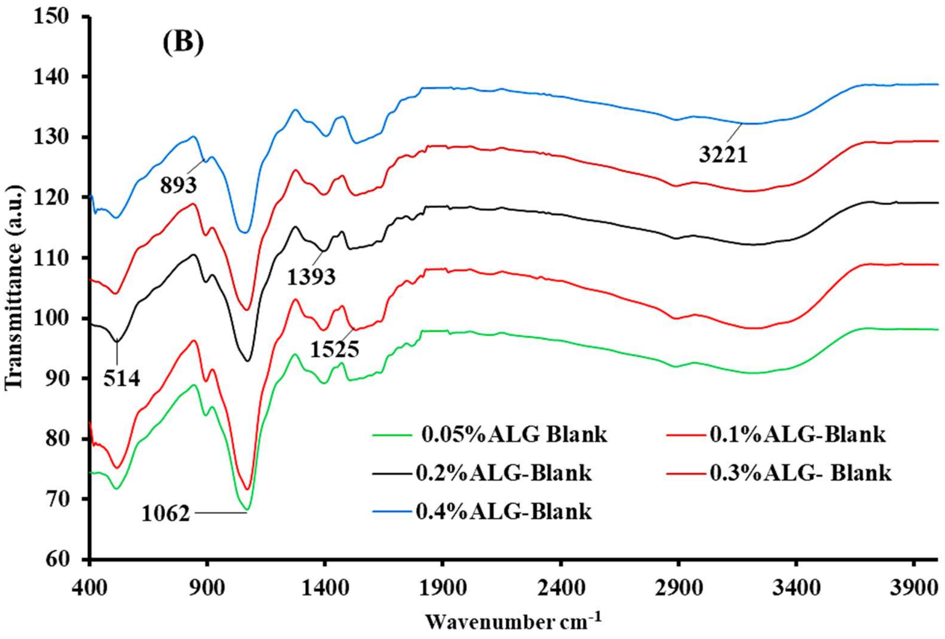


**Figure S2.** FTIR analysis of ALG coated blank chitosan nanoparticles.

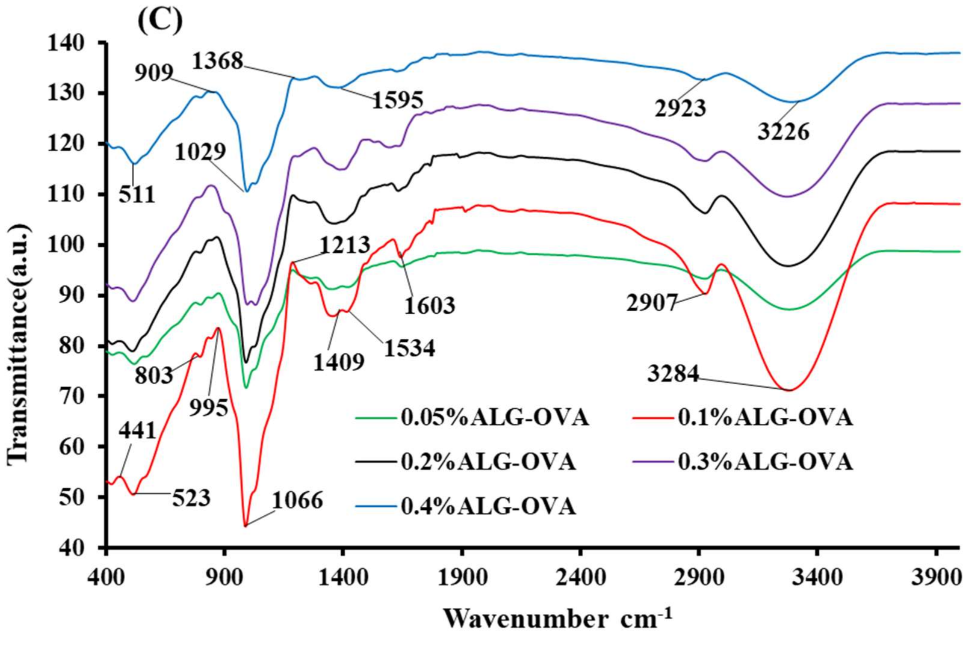


**Figure S3.** FTIR spectra of ALG coated OVA loaded chitosan nanoparticles.

Figures S4 and S5 show FTR spectra of PEG coated blank and OVA loaded chitosan nanoparticles. The results indicate that, PEG coating at concentration of 0.05% showed a sharp peak at 3483 $cm^{-1}$ corresponding to bending of O-H, with stretching N-$CH_3$. PEG 0.1% OVA loaded chitosan nanoparticles showed a band at 2878 $cm^{-1}$, characteristic absorption band at about 1727 $cm^{-1}$ for acetyl C = O, sharp peaks at 1645 and 1568 $cm^{-1}$ represent the furanone C = O and amide C = O groups respectively. FTIR results for coated particles at 0.3% and 0.4% concentration of PEG indicate that the characteristic peaks at

3483 cm$^{-1}$ for OH band has shifted slightly to 3426 and 3392 cm$^{-1}$ respectively due to the presence of amide group from protein.

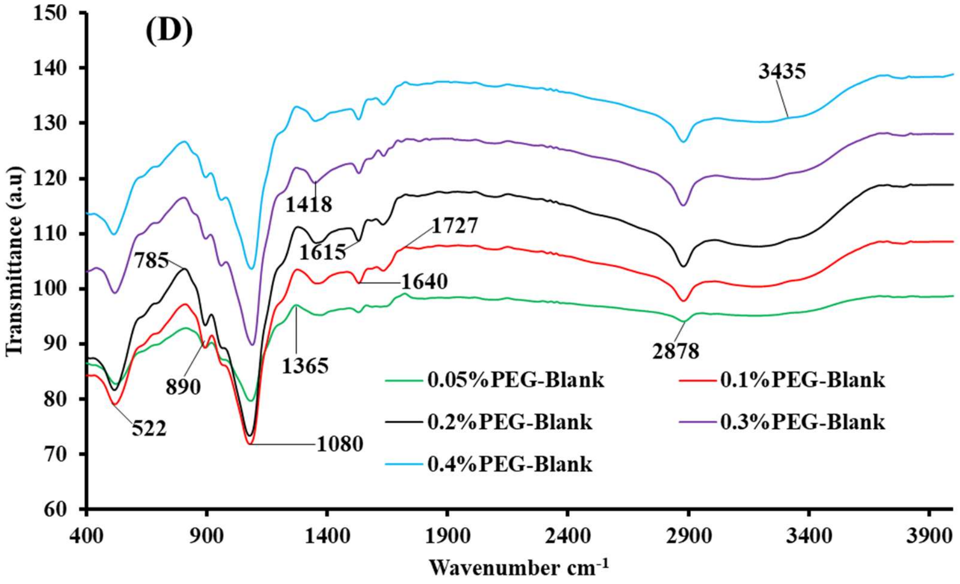


**Figure S4.** FTIR spectra of PEG coated blank chitosan nanoparticles.

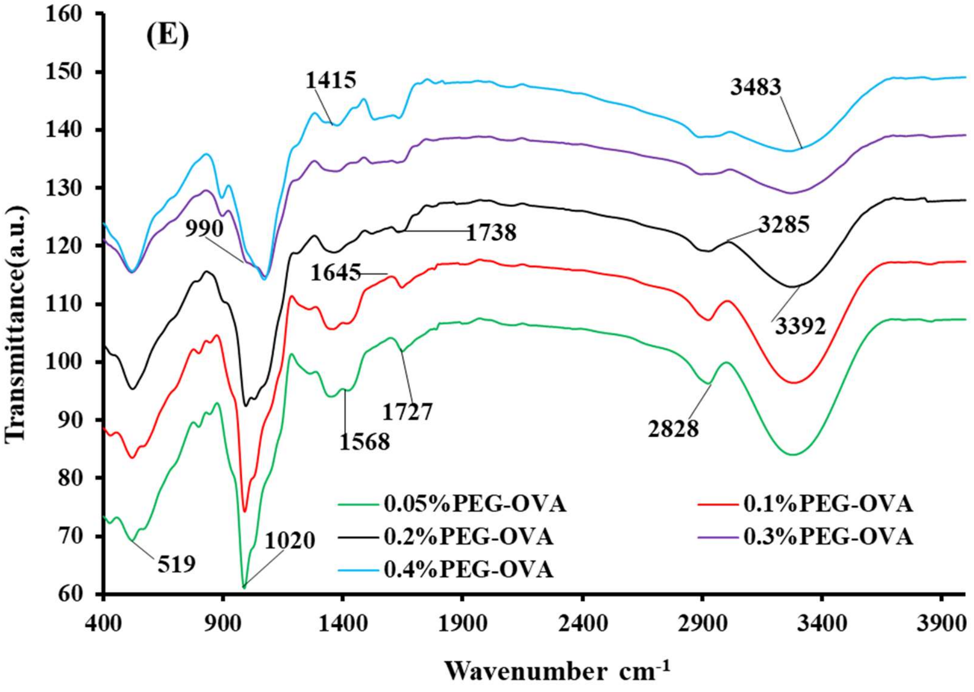


**Figure S5.** FTIR spectra of PEG coated OVA loaded nanoparticles.

**S2 X-ray diffractometry (XRD) analysis.**

X-ray diffraction patterns of pure materials are shown in Figure S6 and the diffractograms for the coated blank and coated OVA loaded chitosan nanoparticles shown in figures S7-S10 respectively. In figure S6, pure PEG and TPP showed sharp crystalline peaks while broad peaks were observed for pure OVA, ALG and PEG diffractogram which are indicative of amorphous nature.

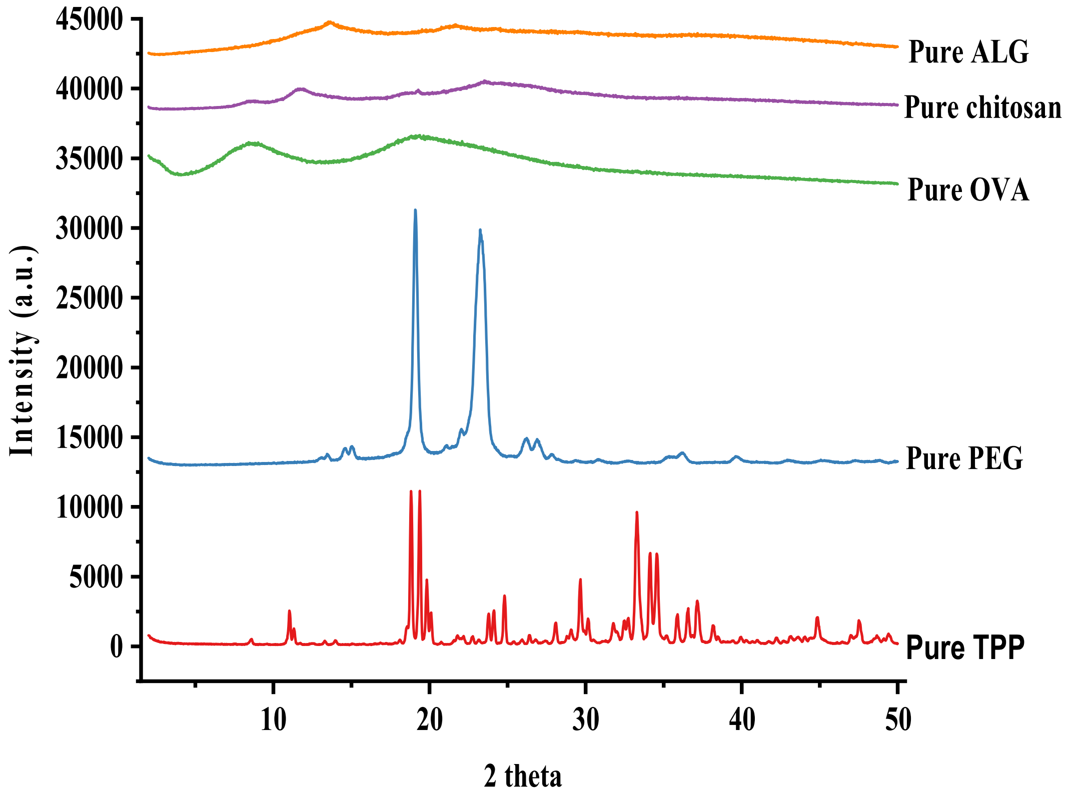


**Figure S6.** XRD diffractograms of pure materials.

X-ray diffractogram in figures S7 and S8, for blank ALG coated and OVA loaded ALG coated nanoparticles exhibited broad peaks, indicating full amorphous nature. XRD diffractogram of pure TPP showed crystalline peaks in figure S6 but was absent in ALG coated chitosan nanoparticles formulation. This is possibly because the colloidal suspension was formed in such a way that the chitosan chains developed intermolecular and extra molecular hydrogen bonds with chain realignment during ionotropic gelation for blank nanoparticles and ultra-sonication during protein loading.

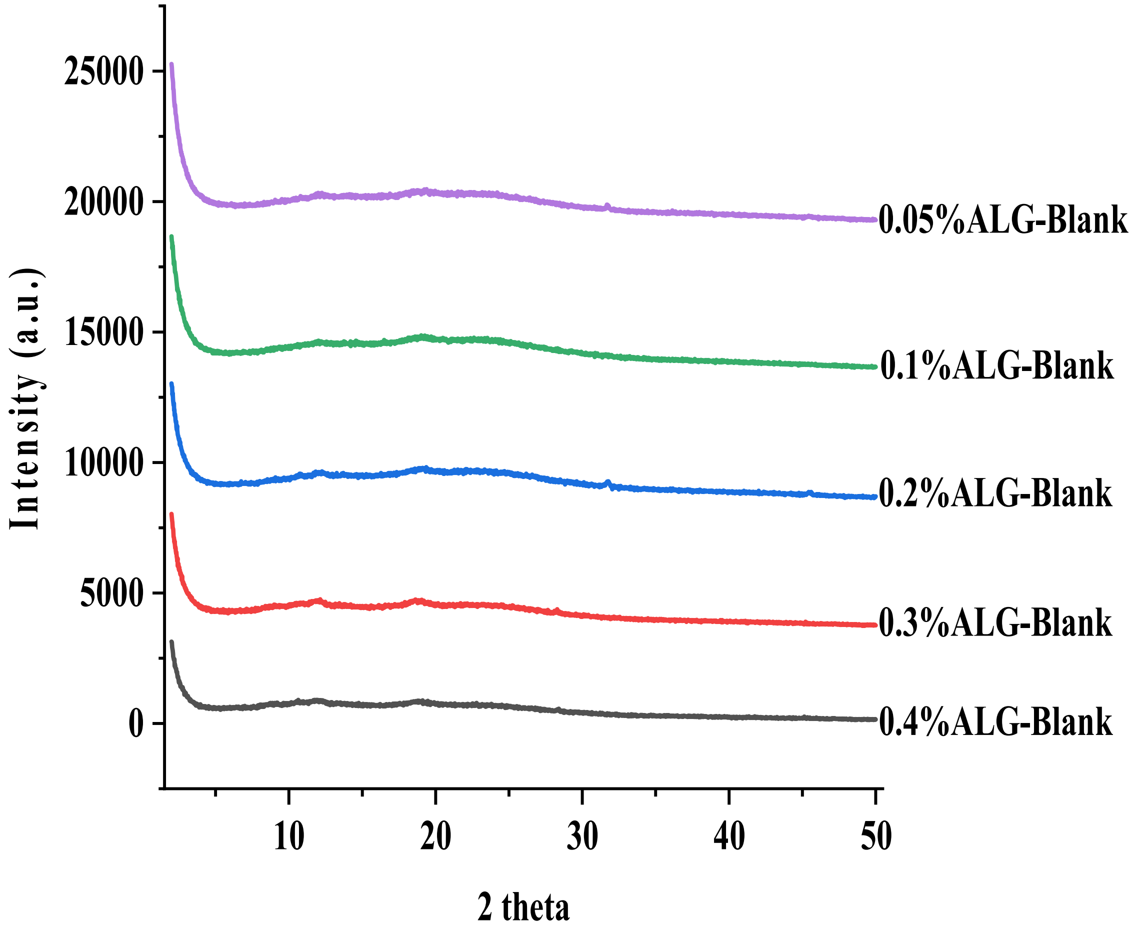


**Figure S7.** X-ray diffractograms of ALG coated blank chitosan nanoparticles.

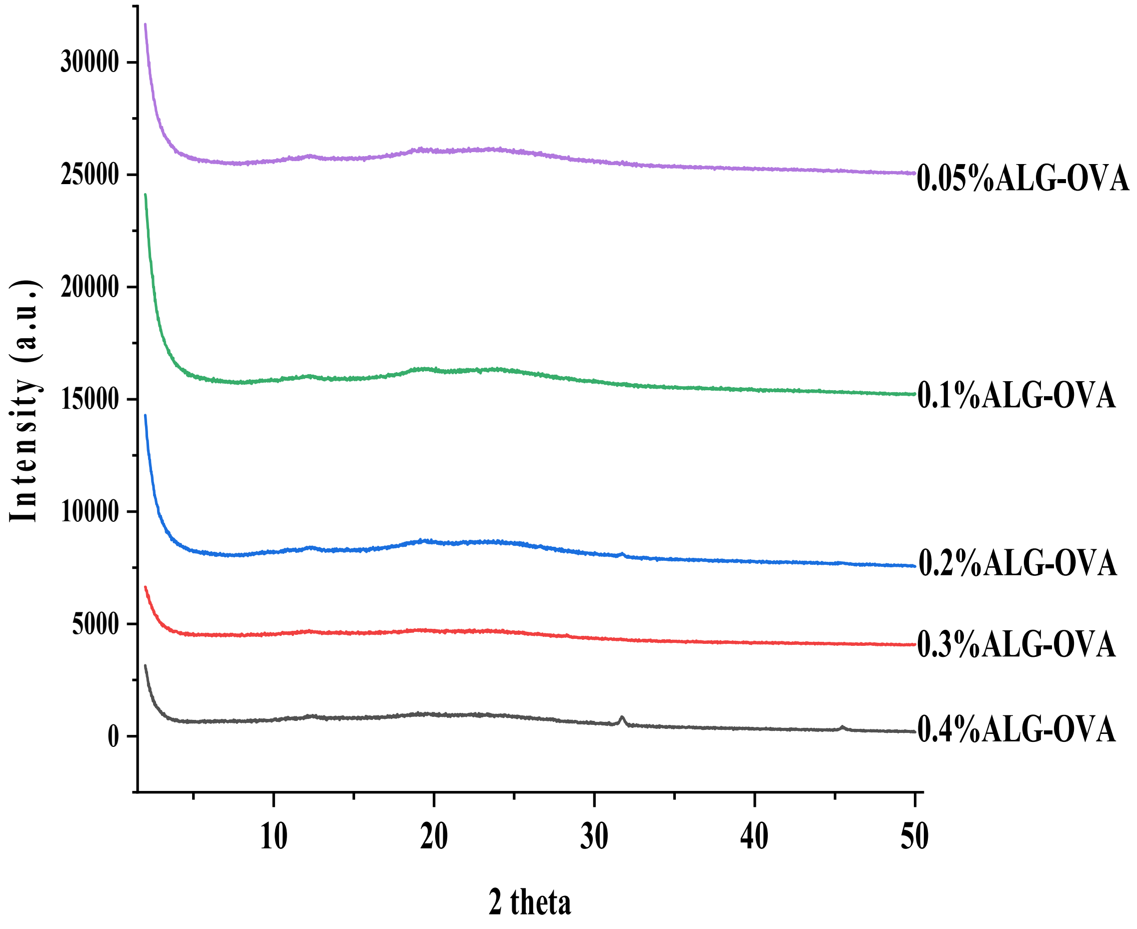


**Figure S8.** X-ray diffractograms of ALG coated OVA loaded chitosan nanoparticles.

On the other hand, in the X-ray diffractogram of pure PEG powder showed several intense peaks observed at 18.50, 22.96, 28.04 and 33.24 2θ, indicating PEG as a crystalline material. In PEG modified blank nanoparticles (figure S9), several intense sharp peaks were observed which suggested that the blank PEG-coated chitosan nanoparticles were crystalline in nature, especially in 0.4% of PEG modified blank nanoparticles. From PEG concentration 0.05% to 0.4% in blank chitosan nanoparticles, crystalline peaks increased as shown in figure S9.

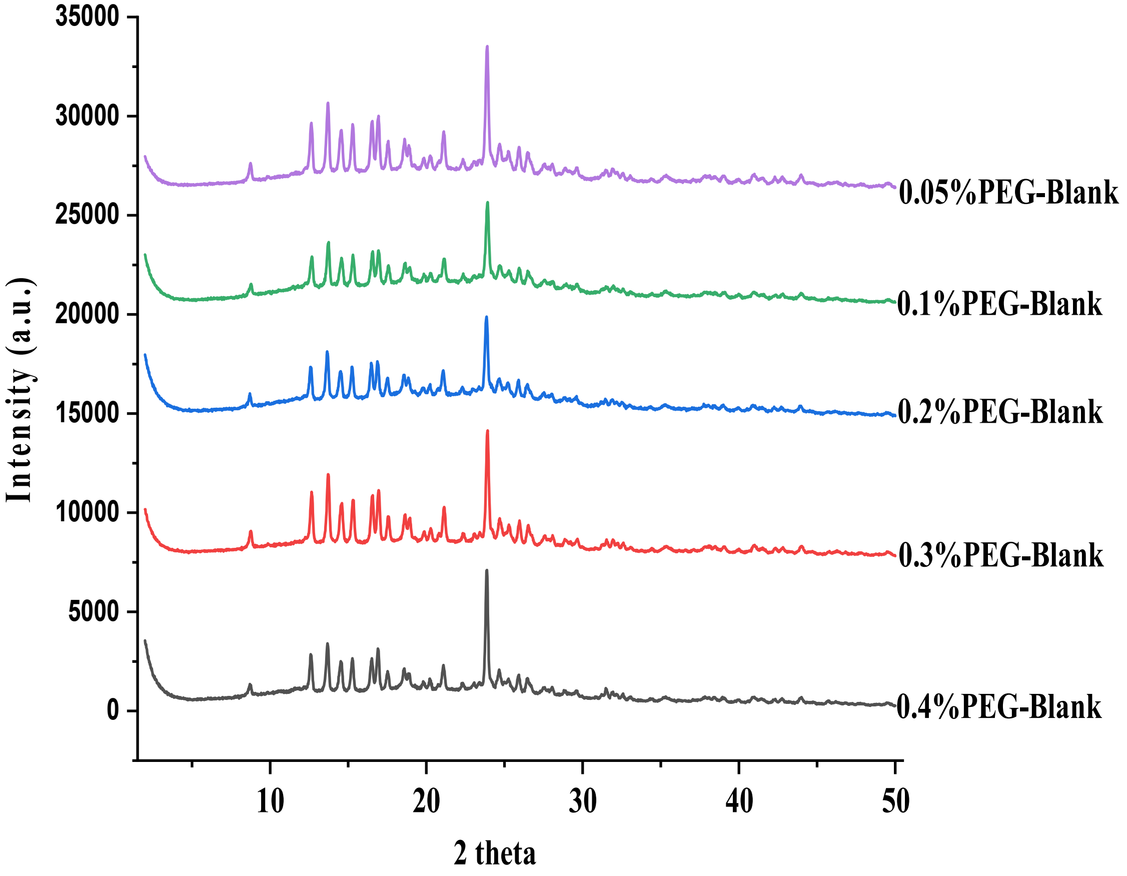


**Figure S9.** X-ray diffractograms of PEG coated blank chitosan nanoparticles.

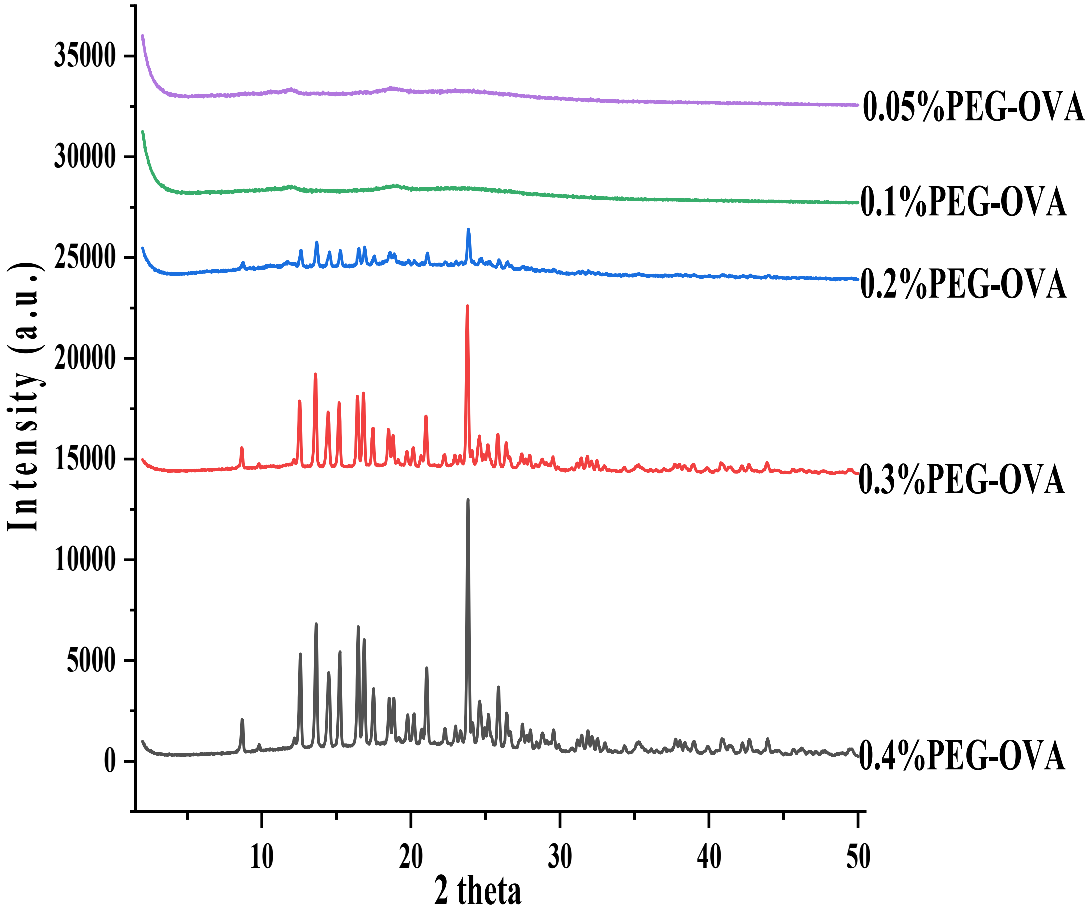


**Figure S10.** X-ray diffractograms of PEG coated OVA loaded chitosan nanoparticles.

This suggests that increased PEG concentration also gradually resulted in increasing crystallinity. Interestingly, after OVA was loaded, PEG modified chitosan nanoparticles at the PEG concentration of 0.05% and 0.1%, showed disappearance of the sharp crystalline peaks or were significantly reduced, which suggested decreased crystallinity after protein loading due to amorphous nature of the OVA (figure S10). This confirms the DSC analysis (section S3 below), where no similar peaks were observed after OVA was loaded into the PEG-modified chitosan nanoparticles formulations. The amorphous phase of the protein and drug loaded nanoparticles has been reported in several research papers [1,2] and results from physical complexation process within the polymer. In the amorphous state of the formulation, molecules are arranged randomly, resulting faster dissolution rate, thereby potentially increasing the protein availability at the target site, however, there is less stability in the amorphous state.

**S3 DSC analysis**

DSC thermograms are shown in figure S11 for pure materials, while figures S12 and S13 show thermograms for ALG coated blank and OVA loaded ALG coated chitosan

nanoparticles. Figures S14 and S15 show the thermograms for PEG coated blank and OVA loaded PEG coated chitosan nanoparticles.

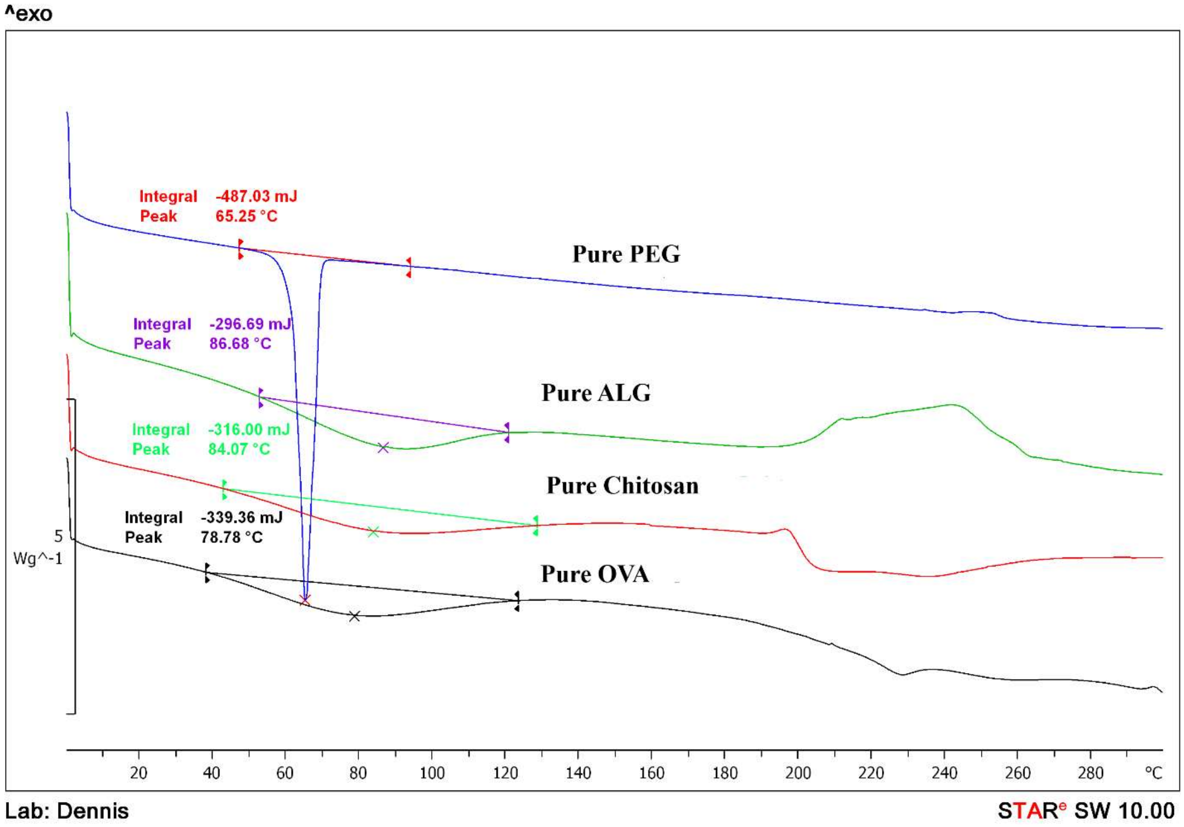


**Figure S11.** DSC thermograms of pure materials.

A sharp endothermic peak was observed for pure PEG at 65.25 °C confirming its crystalline nature. Broad endothermic peaks were observed at 86.68 °C for pure ALG, 84.7 °C for chitosan and 78.78 °C for pure OVA. In figure S12, the nanoparticles prepared with ALG concentration of 0.05% and 0.1% exhibited exothermic glass transition peaks at 143.35 °C and 164.29 °C respectively. Blank 0.2 to 0.4% ALG-chitosan nanoparticles showed endothermic peaks at 85.27 °C, 83.91 °C and 85.25 °C corresponding to loss of bound water from chitosan nanoparticles. Major change was observed after OVA was loaded into the chitosan nanoparticles as these endothermic peaks were not seen compared with blank nanoparticle ALG 0.05 to 0.4% formulations which confirms the XRD results.

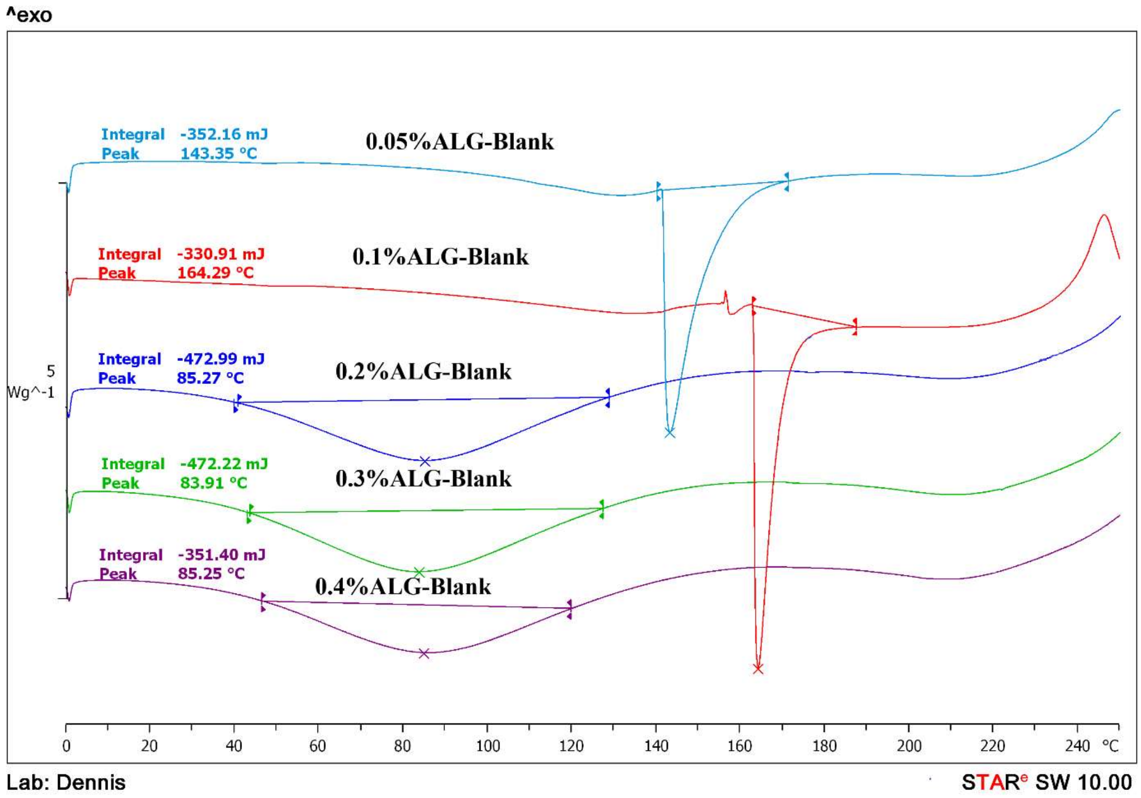


**Figure S12.** DSC thermograms of ALG coated blank nanoparticles.

The DSC thermograms of OVA loaded ALG coated chitosan nanoparticles are shown in figure S13 with glass transition temperature peaks detected between 250 to 260°C. In formulations prepared using 0.05% and 0.1% of chitosan, sharp endothermic peaks were observed in blank nanoparticles which shifted after protein loading. In figure S12 broad peaks were observed around 85°C in 0.2, 0.3 and 0.4% ALG coated blank chitosan nanoparticles which were near 100°C in protein loaded formulations in figure S13. This suggested that protein loading reduced the crystalline state of nanoparticle formulation. The possible reasons include (i) excess amount of unreacted ALG not binding with the dispersed chitosan nanoparticle and rather interacts with protein molecule, (ii) due to electrostatic interaction between negative charge of protein and positive charge of ALG. A similar observation was reported in Juríková and co-worker's study [3].

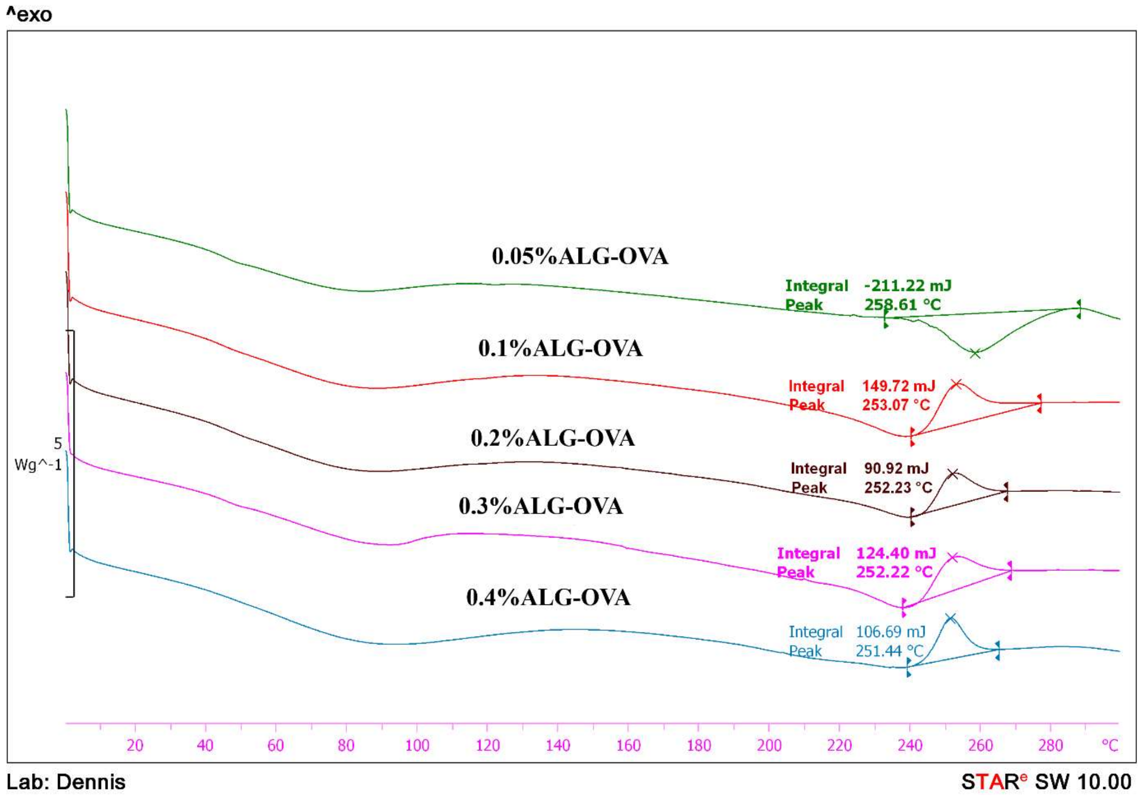


**Figure S13.** DSC thermograms of OVA loaded alginate coated chitosan nanoparticles.

On the other hand, PEG modified chitosan nanoparticles (figure S14) showed a sharp endothermic peak at 58°C for 0.05% PEG coated chitosan nanoparticles. PEG coated blank chitosan nanoparticles (figure S14) exhibited a broad peak in the range of 83-86 °C and absence of peaks was observed in protein loaded formulation in the same range. Compared with pure PEG thermograms where a sharp melting peak was observed at 65.25 °C, the same peak at lower intensity was observed in the blank PEG coated nanoparticles in the range 58-60 °C. The protein loaded formulation showed broad endothermic peaks at 90-100 °C range associated with water loss peaks. The DSC thermograms for PEG coated and OVA loaded chitosan nanoparticles showed broad endothermic peaks at 240-250 °C associated with possible degradation and exothermic peaks 243-253 °C corresponding to polymer chain crystallization process that occurs in conjunction with condensation process. Comparing the thermograms of blank PEG coated nanoparticles (figure S14) with OVA loaded nanoparticles (figure S15), the endothermic peaks were shifted from lower temperature 83- 86°C range to higher temperature 95 to 100°C due to the reduced crystallinity after protein loading.

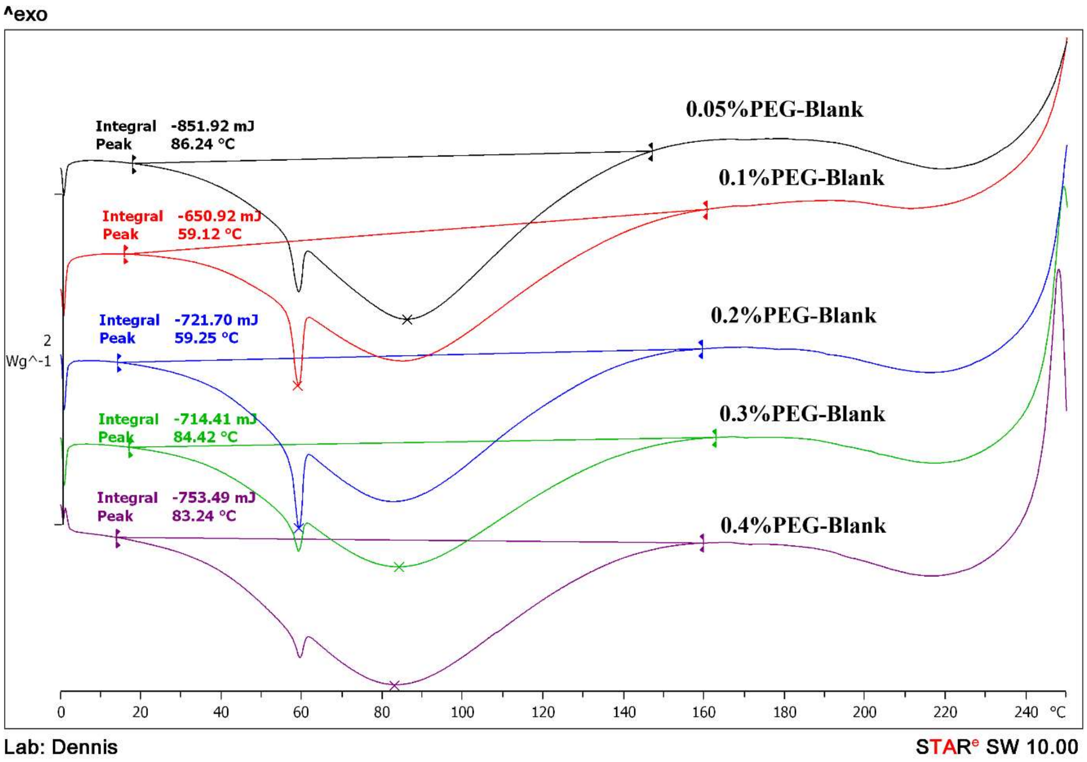


**Figure S14.** DSC thermograms of PEG coated blank chitosan nanoparticles.

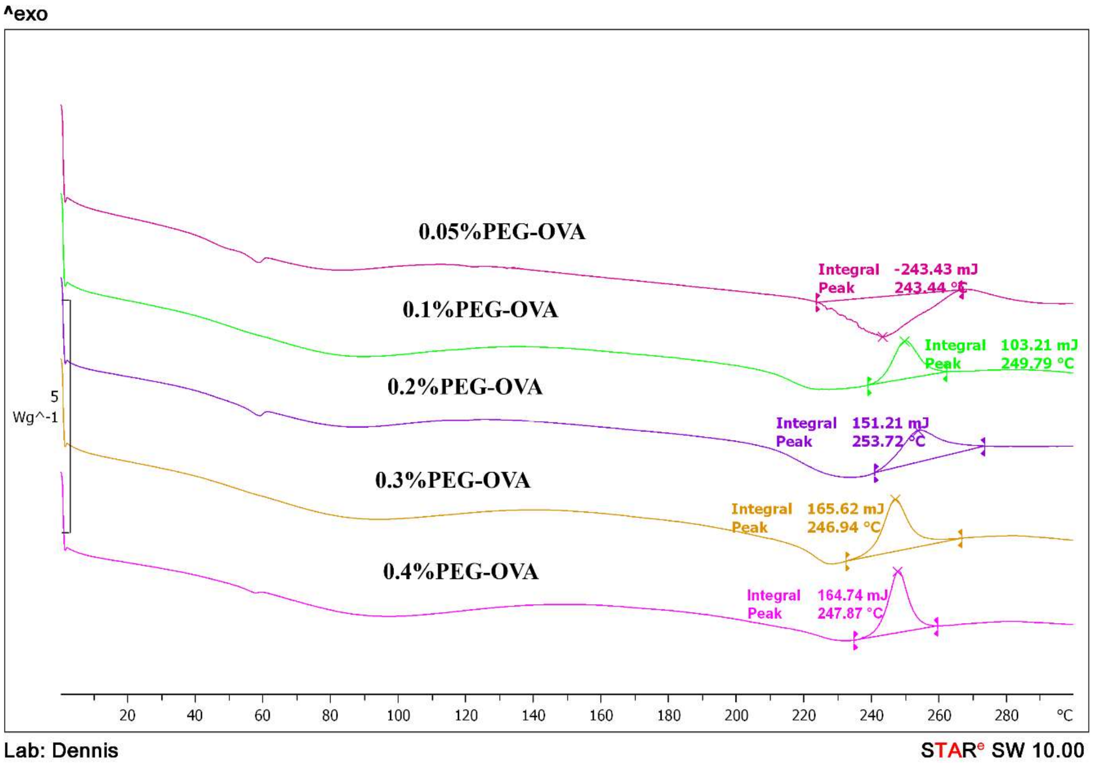


**Figure S15.** DSC thermograms of OVA loaded PEG coated chitosan nanoparticles.

**S4 Scanning electron microscopy and Scanning transmission electron microscopy**

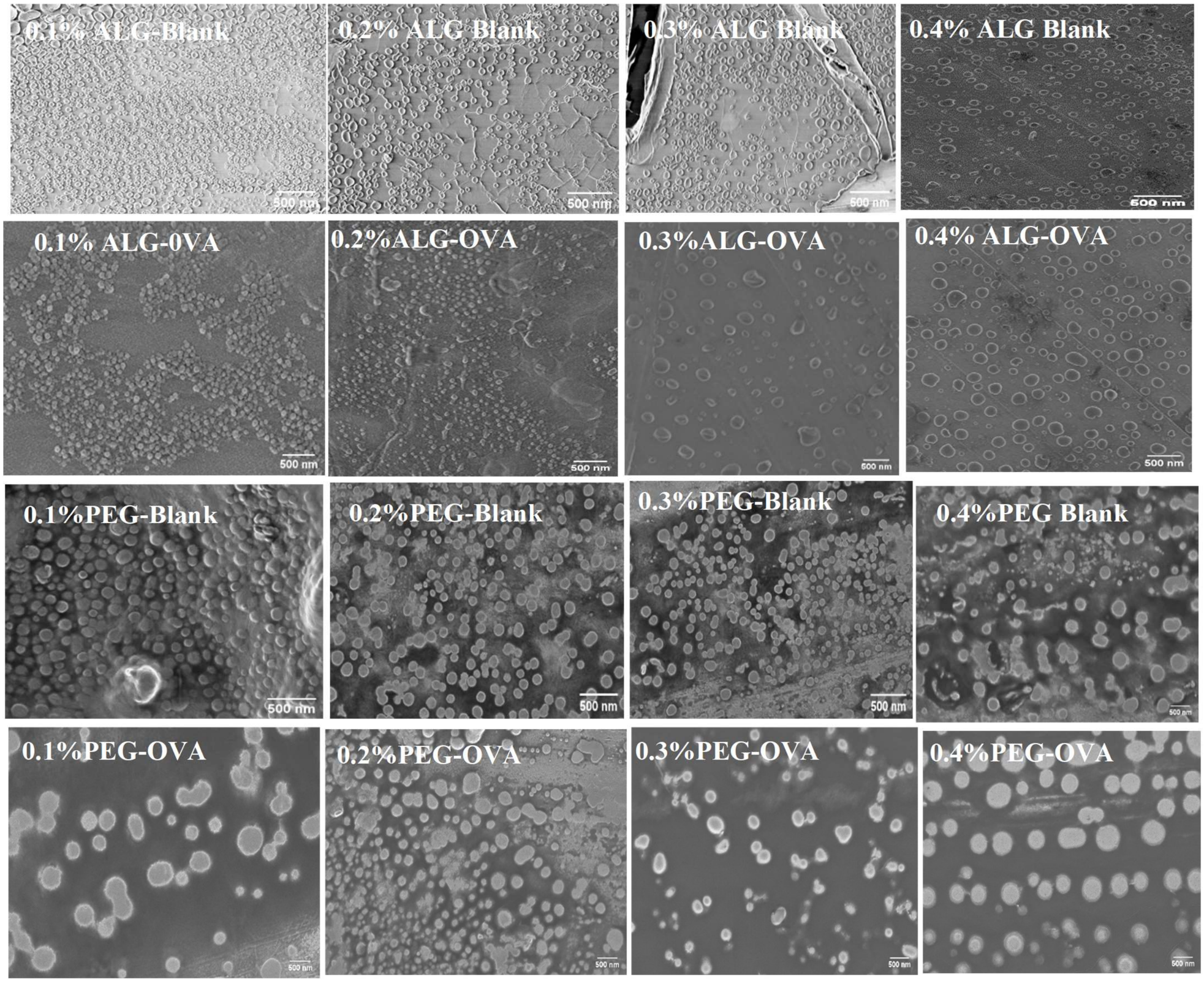


**Figure S16.** SEM images of blank and OVA loaded ALG and PEG coated chitosan nanoparticles with different concentrations of ALG/PEG coating.

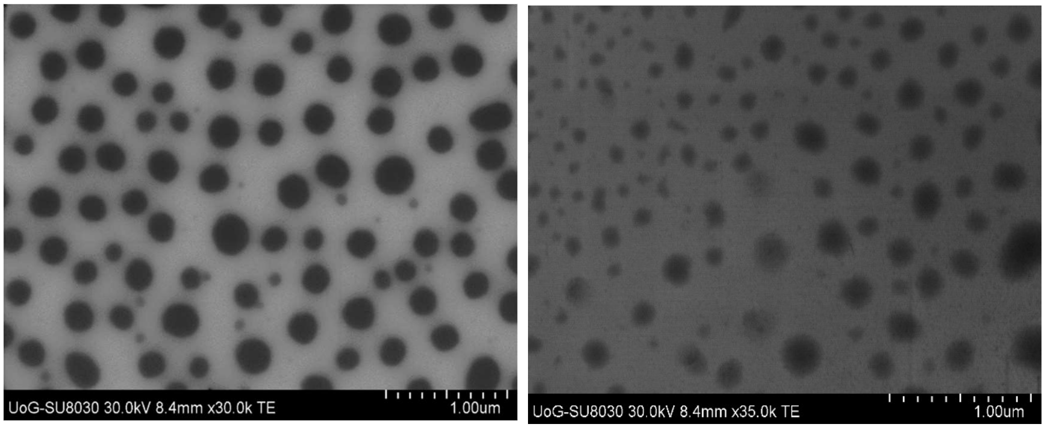


(a) (b)

**Figure S17.** (a) representative STEM image of 0.2%ALG-OVA in simulated gastric fluid after 240 minutes (b) representative STEM image of 0.2%PEG-OVA in simulated gastric fluid after 240 minutes.

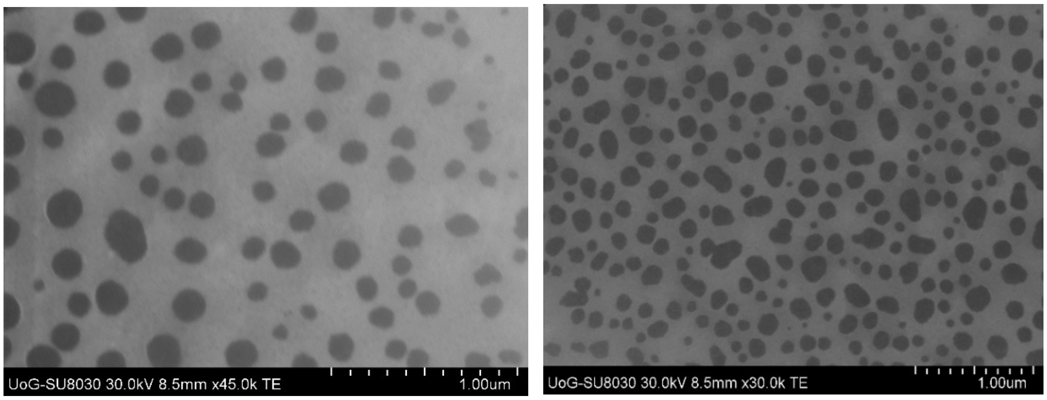


(a) (b)

**Figure S18.** (a) STEM images 0.2%ALG-OVA, sample analyzed after 120 hours in simulated intestinal fluid (b) STEM images of 0.2%PEG-OVA, sample analyzed after 120 hours in simulated intestinal fluid.

## Materials and Methods

### Fourier transform infrared (FTIR) spectroscopy

The infrared spectra were obtained for both pure starting materials and the various nanoparticles at 20 °C for wavenumbers ranging from 4000-400 $cm^{-1}$. FTIR analyses were performed using a Perkin Elmer FT-IR spectrophotometer (Waltham, MA, USA). Different peaks in the IR spectrum were interpreted for different functional groups in the formulations.

### X-ray diffraction (XRD)

X-ray diffraction was performed to investigate crystalline or amorphous nature of the formulated nanoparticles. The diffractograms were recorded using a Brucker AXS D8 Advance instrument (Billerica, MA, USA) with Cu-ka line as a source of radiation and operated at a voltage of 40 keV and current of 35 mA. Freeze dried formulated samples

and pure starting materials were measured between 3º and 80º 2θ for 2 hours and step time of 5 seconds.

**Differential scanning calorimetry**

Differential scanning calorimetry (Mettler Toledo 823e, Greifensee, Switzerland)) was performed to characterize the physical state of lyophilized chitosan nanoparticles. About 2.5 – 3 mg of accurately weighed sample was placed into aluminium pans with lids and analyzed at a scanning temperature range from 0 to 300 °C and heating rate of 10 °C/min under continuous stream of dry nitrogen gas. In addition, pure starting materials were also analyzed and compared with the formulated nanoparticles.

## References

1. Rajan, M.; and Raj, V. Encapsulation, characterisation and in-vitro release of anti-tuberculosis drug using chitosan - poly ethylene glycol nanoparticles. *Int. J. Pharm. Pharm. Sci*. **2012**, *4(4)*, 255-259. doi. 10.1002/slct.201701396
2. Pelaz, B., Pino, P.D.; Maffre, P.; Hartmann, R.; Gallego, M.; Rivera- Fernández, S.; Fuente, J.M.D.L.; Nienhaus, G.U.; and Parak, W.J. Surface Functionalization of nanoparticles with polyethylene glycol: effects on protein adsorption and cellular uptake. *ACS Nano*. **2015**, *9*, 6996-7008. doi: 10.1021/acsnano.5b01326.
3. Juríková, A.; Csach, K.; Miškuf, J.; Koneracká, M.; Závišová, V.; Kubovčíková, M.; Kopčanský, P.; and Múčková, M. Thermal properties of magnetic nanoparticles modified with polyethylene glycol. *IEEE Transact. Magnet*. **2013**, *49(1)*, 236-239, doi: 10.1109/TMAG.2012.2224322.
